# Neuronal cathepsin S increases neuroinflammation and causes cognitive decline via CX3CL1‐CX3CR1 axis and JAK2‐STAT3 pathway in aging and Alzheimer's disease

**DOI:** 10.1111/acel.14393

**Published:** 2024-10-25

**Authors:** Pei‐Pei Liu, Xiao‐Hui Liu, Ming‐Jing Ren, Xiao‐Tong Liu, Xiao‐Qing Shi, Ming‐Li Li, Shu‐Ang Li, Yang Yang, Dian‐Dian Wang, Yue Wu, Fan‐Xiang Yin, Yan‐Hong Guo, Run‐Zhou Yang, Meng Cheng, Yong‐Juan Xin, Jian‐Sheng Kang, Bing Huang, Kai‐Di Ren

**Affiliations:** ^1^ Clinical Systems Biology Laboratories The First Affiliated Hospital of Zhengzhou University Zhengzhou Henan China; ^2^ Department of Neurology The First Affiliated Hospital of Zhengzhou University Zhengzhou Henan China; ^3^ Department of Nephropathy The First Affiliated Hospital of Zhengzhou University Zhengzhou Henan China; ^4^ Department of Clinical Laboratory The First Hospital of Yongnian District Hebei China; ^5^ Department of Clinical Laboratory The First Affiliated Hospital of Zhengzhou University Zhengzhou Henan China; ^6^ Translational Medical Center The First Affiliated Hospital of Zhengzhou University Zhengzhou China; ^7^ Henan Branch Bank of China Zhengzhou Henan China; ^8^ Department of Child and Adolescent Health Precision Nutrition Innovation Center, School of Public Health, Zhengzhou University Zhengzhou Henan China; ^9^ Pain and Related Disease Research Laboratory Shantou University Medical College Shantou Guangdong China; ^10^ Department of Pharmacy The First Affiliated Hospital of Zhengzhou University Zhengzhou Henan China

**Keywords:** aging, Alzheimer's disease, cathepsin S, neurodegenerative disease, neuroinflammation, recognition

## Abstract

Aging is an intricate process involving interactions among multiple factors, which is one of the main risks for chronic diseases, including Alzheimer's disease (AD). As a member of cysteine protease, cathepsin S (CTSS) has been implicated in inflammation across various diseases. Here, we investigated the role of neuronal CTSS in aging and AD started by examining CTSS expression in hippocampus neurons of aging mice and identified a significant increase, which was negatively correlated with recognition abilities. Concurrently, we observed an elevation of CTSS concentration in the serum of elderly people. Transcriptome and fluorescence‐activated cell sorting (FACS) results revealed that CTSS overexpression in neurons aggravated brain inflammatory milieu with microglia activation to M1 pro‐inflammatory phenotype, activation of chemokine C‐X3‐C‐motif ligand 1 (CX3CL1)—chemokine C‐X3‐C‐motif receptor 1 (CX3CR1) axis and janus kinase 2 (JAK2)—signal transducer and activator of transcription 3 (STAT3) pathway. As CX3CL1 is secreted by neurons and acts on the CX3CR1 in microglia, our results revealed for the first time the role of neuron CTSS in neuron–microglia “crosstalk.” Besides, we observed elevated CTSS expression in multiple brain regions of AD patients, including the hippocampus. Utilizing CTSS selective inhibitor, LY3000328, rescued AD‐related pathological features in *APP/PS1* mice. We further noticed that neuronal CTSS overexpression increased cathepsin B (CTSB) activity, but decreased cathepsin L (CTSL) activity in microglia. Overall, we provide evidence that CTSS can be used as an aging biomarker and plays regulatory roles through modulating neuroinflammation and recognition in aging and AD process.

## Graphical abstract

Showing the roles of neuronal CTSS in the process of aging and Alzheimer's disease (AD). As a cysteine protease cathepsin family member, CTSS degrades proteins along the endocytic pathway in young and healthy mice. In aging and AD model mice, the expression level of neuronal CTSS is significantly elevated in neurons, which increases neuroinflammation and causes cognitive decline via CX3CL1‐CX3CR1 axis and JAK2‐STAT3 pathway. LY 3000328, the selective inhibitor of CTSS, LY3000328, significantly rescues AD‐related pathological features in APP/PS1 mice.

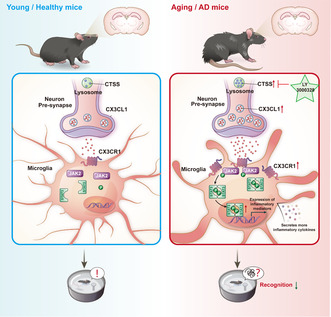

**Abbreviations**
AAV: adeno‐associated virus
AD: Alzheimer's disease
ALS: amyotrophic lateral sclerosis
Aβ: β‐amyloid
CCR: chemokine (C‐C motif) receptor
CNS: central nervous system
Cox‐2: cyclooxygenase‐2
CTSB: cathepsin B
CTSL: cathepsin L
CTSS: cathepsin S
CX3CL1: chemokine C‐X3‐C‐motif ligand 1
CX3CR1: chemokine C‐X3‐C‐motif receptor 1
DEGs: differentially expressed genes
DG: dental gyrus
DMSO: dimethyl sulfoxide
ELISA: enzyme‐linked immunosorbent assay
GO: gene Ontology
GSEA: gene set enrichment analysis
HD: Huntington's disease
IFN: interferon
IL: interleukin
Irf8: interferon regulatory factor 8
JAK2: Janus kinase 2
MHC‐II: major histocompatibility complex II
MWM: Morris's water maze
NC: negative control
PCA: principal component analysis
PD: Parkinson's disease
Runx1: runt‐related transcription factor 1
STAT3: signal transducer and activator of transcription 3
TNF‐α: tumor necrosis factor‐α
TPM: transcripts per million
TREM2: triggering receptor also expressed on myeloid cells‐2
UN: United Nations

## 1 INTRODUCTION

Aging is an intricate process influenced by a multifaceted interplay of genetic, epigenetic, and inflammatory factors (Gaspar‐Silva et al., 2023). This process is characterized by deterioration in the physiological functions of organisms and is closely linked to age‐related chronic diseases (Cai et al., 2022). The central nervous system (CNS) is affected by aging similar to other organs (Hou et al., 2019). Notably, neuronal cells are more susceptible to accumulating damaged proteins, increased oxidative stress, perturbed energy homeostasis, and nucleic acid mutations than other cell types (Sikora et al., 2021). In humans, aging of the nervous system compromises behavioral and cognitive functions. It predisposes to neurodegenerative diseases, including Parkinson's disease (PD), Alzheimer's disease (AD), Huntington's disease (HD), diabetic neuropathy, and amyotrophic lateral sclerosis (ALS) (Wyss‐Coray, 2016), which impose substantial burdens on families and society. Aging is believed to be governed by genetic networks, signaling pathways, and cellular metabolic responses, which build and maintain an aged state (Morris et al., 2019), (Ding et al., 2021). Intervening with key biological mechanisms during aging is a potentially effective strategy to slow or prevent the development of these diseases.

As a cysteine protease cathepsin family member, cathepsin S (CTSS) is vital in protein degradation within the endocytic pathway. Unlike other cysteine cathepsins that require an acidic environment, CTSS can be activated at neutral and acidic pH values (Wilkinson et al., 2015). Due to its better conformational stability and ability to function at neutral pH, CTSS can work in the extracellular environment, degrading extracellular matrix proteins (Gupta et al., 2008). Previous studies have also demonstrated that CTSS is involved in major histocompatibility complex II (MHC‐II) antigen processing and presentation, indicating its involvement in the pathogenesis of immune‐related diseases (McKelvey et al., 2022) (Montague‐Cardoso & Malcangio, 2020). Notably, studies illustrated that CTSS is predominantly expressed in the microglial population of the nervous system (Pišlar & Kos, 2014). CTSS processes microglial MHC‐II to help microglia capture antigenic fragments, but growing data suggest that it may be crucial for the neurotoxic microglial response to neuropathic conditions (Nakanishi, 2020). Furthermore, increased CTSS activity has been observed during aging‐related neurodegenerative processes (Lowry & Klegeris, 2018; Wendt et al., 2008), AD (Lemere et al., 1995), and ALS (Chiu et al., 2013). However, the mechanisms by which neuron CTSS controls aging and AD in animal models and humans remain poorly understood.

Increasing evidence indicates that inflammation is a hallmark of numerous acute and chronic brain disorders, particularly in AD (Akiyama, Barger, et al., 2000); (Agrawal et al., 2022). Microglia are the major resident immune cells in the brain, where they constantly survey the microenvironment and produce factors that affect astrocytes and neurons (Nayak et al., 2014), (Streit, 2002). In AD, inflammatory responses include alterations in microglial morphology—from ramified (resting) to amoeboid (active)—and astrogliosis, characterized by an increase in the number, size, and motility of astrocytes around senile plaques (Kettenmann et al., 2011), (Das & Chinnathambi, 2019). Microglia around plaques exhibit positive staining for activation markers and proinflammatory mediators, including MHC‐II, cyclooxygenase‐2 (Cox‐2), monocyte chemoattractant protein‐1 (MCP‐1), tumor necrosis factor‐α (TNF‐α), interleukin (IL)‐1β, and IL‐6 (Akiyama, Arai, et al., 2000). Additionally, elevated levels of chemokines, cytokines, and their receptors have been reported in post‐mortem AD brains (Cartier et al., 2005). Notably, the chemokine C‐X3‐C‐motif ligand‐chemokine C‐X3‐C‐motif receptor 1 (CX3CL1‐CX3CR1) axis up‐regulation is consistently associated with spanning ischemia, AD, PD, and HD, implying its crucial role in the neurodegenerative processes (Subbarayan et al., 2022). CX3CL1—also known as fractalkine or neurotactin—is constitutively produced by neurons and binds to its receptor CX3CR1 in microglia (Hatori et al., 2002), (Mecca et al., 2018). The CX3CL1‐CX3CR1 axis extends its involvement over numerous inflammatory processes by communicating with diverse inflammatory signaling pathways, including the JAK–STAT, Toll‐like receptor, MAPK, AKT, NF‐κb, and Wnt/β‐catenin pathways (Zhuang et al., 2019), (Harrison et al., 1998).

In our study, we investigated the role of neuron CTSS in regulating recognition behavior in aging and AD model mice and the underlying mechanism. We observed that CTSS expression is robustly upregulated in aging individuals and AD patients, both in humans and rodents, which was negatively correlated with recognition abilities. Neuron CTSS overexpression aggravates brain inflammatory milieu and increases neuron–microglia “crosstalk.” Furthermore, utilizing selective CTSS inhibitor, LY3000328, significantly rescues AD‐related phenotypes of *APP/PS1* mice. Lastly, we detected the activity changes of other cysteine cathepsins due to neuronal CTSS overexpression, and find that cathepsin B (CTSB) activity was increased and cathepsin L (CTSL) activity was decreased in microglia. In conclusion, our study provides the evidence that CTSS can be used as a biomarker for aging and a therapeutic target for AD.

## 2 METERIALS AND METHODS

### 2.1 Animals

All male mice were bred on a C57BL/6J background. B6‐Tg (PrP‐hAPP/hPS1) mice were obtained from Beijing HFK Bioscience Co. Ltd. Mice were housed under specific pathogen‐free conditions with sufficient food and water. Animal experimentation was performed following the law and with permission from local authorities.

### 2.2 Human serum samples

All human serum samples were obtained from the First Affiliated Hospital clinical laboratory of Zhengzhou University with appropriate ethics committee approval (NO. 2023‐KY‐1406). Forty healthy male volunteers participated in our study on November 1st, 2023. We divided the volunteers into the young group (*n* = 20, mean age 31.80 ± 3.12 years) and the aging group (*n* = 20, mean age 66.90 ± 2.86 years). Two exclusion criteria were applied for participants: those who (1) were taking medication or undergoing hospital treatment and (2) had neurological disorders like cognitive dysfunction whereby they were unable to communicate. The baseline characteristics of the 40 volunteers are presented in Table 1.

**TABLE 1:** Baseline characteristics of young and aging volunteers.

Variables	Young	Aging	*p*‐value
*Sex*			
Male	20/20 (100%)	20/20 (100%)	‐
Female	0/20 (0%)	0/20 (0%)	‐
Age (yr) (mean ± SD)	31.80 ± 3.12	66.90 ± 2.86	< 0.001

### 2.3 Enzyme‐linked immunosorbent assay (ELISA)

The CTSS concentration in the serum of patients was evaluated using a human CTSS ELISA kit (Elabscience, #E‐EL‐H5431c, Wuhan, China) following the manufacturer's guidelines. Besides, the CX3CL1 concentration in the medium was evaluated using a mouse CX3CL1 ELISA kit (Elabscience, #E‐EL‐M0267) following the manufacturer's guidelines.

### 2.4 Hippocampus neurons isolation and culture

Primary hippocampus neurons were dissected from postnatal day 1 C57BL/6 mice pups and cultured as described previously (Kang et al., 2008). Briefly, after digestion with trypsin and triturating with fire‐polish pipettes, the hippocampus cells recovered by centrifugation were plated onto 12 mm coverslips. After 2 hours, 2 mL of plating medium was added to each 35 mm dish [For 100 mL of plating medium: 89 mL of Minimal Essential Medium (MEM, Invitrogen), 0.5 g of glucose, 0.5 mM glutamine, 2 g of NaHCO_3_, 10 mg of bovine transferrin (Calbiochem), 2.5 mg of insulin, 10% fetal calf serum (FBS, Gibco)]. From the second day in culture, half of the medium was replaced with feeding medium twice a week [100 mL of feeding medium: 97 mL minimal essential medium, 0.5 g of glucose, 0.5 mM glutamine, 2 g of NaHCO_3_, 10 mg of bovine transferring, 3 mM cytosine‐p‐arabinofuranoside (Sigma‐Aldrich) and 2% B27 medium supplement (Invitrogen)]. A density (6000–8000 cells per cm^2^) of neurons was cultured on Matrigel (BD Biosciences) coated coverslips from hippocampi of postnatal day 1 mice pups.

### 2.5 Cell line culture

The murine BV2 microglial cells and HT‐22 hippocampus neurons were cultured in Dulbecco's modified Eagle's medium (DMEM, Gibco) supplemented with 10% FBS and 1% penicillin/streptomycin in 5% CO_2_ at 37°C.

### 2.6 Immunofluorescence

Sections (30 μm) of mouse brains were cut using a cryostat (CM3050S, Leica) and stored at −20°C in cryoprotective storage solution (30% sucrose, 1% polyvinylpyrrolidone, 5 mM Na2HPO4, 20 mM NaH2PO4, and 30% ethylene glycol) until use. The sections were washed three times with phosphate‐buffered saline (PBS) and incubated with blocking buffer (0.3% Triton‐X‐100 and 5% goat serum in PBS) for 1.5 h at room temperature, followed by incubation with primary antibodies overnight at 4°C. Primary antibodies against CTSS (1:500, Santa Cruz, sc‐74,429, California, USA), NeuN (1:500, Millipore, #Mab377, Massachusetts, USA), Iba1 (1:300, Abcam, ab178846, Cambridge, UK), Iba1 (1:300, Abcam, ab5076), 6E10 (1:500, Biolegend, #803014, San Diego, California, USA), beta Amyloid 1–42 (1:300, Abcam, ab201060, Cambridge, UK), CX3CR1 (1:500, Thermo Fisher, 14–7986‐81, Waltham, Massachusetts, USA), CX3CR1 (1:300, Abcam, ab308613), CX3CL1 (1:500, Thermo Fisher, 14–6093‐81), cAMP response element‐binding protein (CREB) (1:300, CST, #9197, Danvers, Massachusetts, USA), and p‐CREB (1:300, CST, #9198) were applied, followed by washes in 1 × PBS and visualization with Alexa 488‐, 546‐, or 633‐conjugated secondary antibodies (Invitrogen, Carlsbad, California, USA). 4′, 6‐Diamidino‐2‐phenylindole (DAPI) (1:10^6^, CST, #4083) was used to stain the nuclei.

### 2.7 Image acquisition and analysis

The stained specimens were imaged using a Zeiss 980 confocal microscope. The colocalization of CTSS and NeuN was imaged with a 60×/1.4 objective (Zeiss, Oberkochen, Germany). The colocalization of CTSS and 6E10 was imaged with a 20×/0.95 objective (Zeiss) and a 60x/1.41 objective (Zeiss). The number of Iba1 positive cells was determined using a 20×/0.95 objective (Zeiss). The colocalization of EGFP, CTSS, and DAPI; GFP, CX3CL1, and DAPI; and GFP, CX3CR1, Iba1, and DAPI were imaged with a 20×/0.95 objective (Zeiss). The 6E10 plaques staining were imaged with a 10×/0.45 objective (Zeiss). Briefly, 8–9 mice from each group were analyzed. Image processing was performed using ImageJ analysis software (Version 1.49; NIH, Bethesda, Maryland, USA) and was limited to rotation, cropping, uniform brightness and contrast adjustments, and maximum intensity projection of the volumes.

### 2.8 Immunoblot analysis

Total proteins were extracted from the tissue and cells using radioimmunoprecipitation assay buffer (CST, #9806) supplemented with protease and phosphatase inhibitors (Thermo Fisher, #78441). The protein concentrations of the samples were determined by a microplate bicinchoninic acid protein assay kit (Beyotime Biotechnology, Shanghai, China). Equal amounts of protein (30 μg) were subjected to a 12% sodium dodecyl sulfate‐polyacrylamide gel electrophoresis and transferred onto a nitrocellulose membrane for Immunoblot analysis. Antibodies used for Immunoblot include the following: CTSS (1:1000, Santa Cruz, sc‐74,429), glyceraldehyde‐3‐phosphate dehydrogenase (GAPDH) (1:5000, Proteintech, 60,004‐1‐Ig, Rosemont, Illinois, USA), β‐actin (1:5000, Proteintech, 81,115‐1‐RR), CX3CR1 (1:1000, Thermo Fisher, 14–7986‐81), CX3CL1 (1:1000, Thermo Fisher, 14–6093‐81), JAK2 (1:1000, CST, #3230), p‐JAK2 (1:1000, CST, #3771), STAT3 (1:1000, CST, #9139), p‐STAT3 (1:1000, CST, #9145), cathepsin B (1:1000, Abcam, ab214428), and cathepsin L (1:1000, Santa Cruz, sc‐390,367). Blots were visualized using an LI‐COR Odyssey imager, and the ImageJ analysis software (Version 1.49; NIH, USA) was used to determine each band intensity. Band densities of the indicated proteins were normalized to those of the corresponding loading controls.

### 2.9 RNA isolation and quantitative real‐time polymerase chain reaction (qRT‐PCR)

Tissue and cells were lysed using a Total RNA Isolation Reagent (TRIzol reagent) (Invitrogen). Total RNA was extracted following the manufacturer's protocol. RNA was then analyzed for purity by measuring the absorbance ratio at 260 and 280 nm using the NanoPhotometer Spectrometer (Thermo Fisher). Following the manufacturer's protocol, cDNA libraries were synthesized using One‐Step gDNA Removal and cDNA Synthesis SuperMix (Takara, Kyoto, Japan). A reverse transcription‐polymerase chain reaction (RT‐PCR) assay was conducted using 2× TB Green qPCR Master Mix (Takara), and the threshold cycle (Ct) value was measured. GAPDH was used as the housekeeping gene for normalization, respectively. The comparative gene expression was calculated with the 2 − ΔΔCt method. All primers were synthesized by Genewiz Biotech (Nanjing, China). The primers used are listed in Table 2.

**TABLE 2:** Primers sequences.

Genes	Primers sequences
*Gapdh*‐forward	ATGTGTCCGTCGTGGATCTGA
*Gapdh*‐reverse	ATGCCTGCTTCACCACCTTCT
*ctss*‐forward	GTGGCCACTAAAGGGCCTG
*ctss*‐reverse	ACCGCTTTTGTAGAAGAAGAAGGAG
*gfap*‐forward	TCCTGGAACAGCAAAACAAG
*gfap*‐reverse	CAGCCTCAGGTTGGTTTCAT
*Iba* 1‐forward	GTCCTTGAAGCGAATGCTGG
*Iba*1‐reverse	CATTCTCAAGATGGCAGATC
*cx3cl1*‐forward	ACGAAATGCGAAATCATGTGC
*cx3cl1*‐reverse	CTGTGTCGTCTCCAGGACAA
*cx3cr1*‐forward	CAGCATCGACCGGTACCTT
*cx3cr1*‐reverse	GCTGCACTGTCCGGTTGTT
*Cd11b*‐forward	CCTTCATCAACACAACCAGAGTGG
*Cd11b*‐reverse	CGAGGTGCTCCTAAAACCAAGC
*Cd68*‐forward	TTCACCTTGACCTGCTCTCTC
*Cd68*‐reverse	GTAGGTTGATTGTCGTCTGCG
*Cd3*‐forward	ATGCGGTGGAACACTTTCTGG
*Cd3*‐reverse	GCACGTCAACTCTACACTGGT
*Cd4*‐forward	AGGTGATGGGACCTACCTCTC
*Cd4*‐reverse	GGGGCCACCACTTGAACTAC
*Cd8a*‐forward	CCGTTGACCCGCTTTCTGT
*Cd8a*‐reverse	CGGCGTCCATTTTCTTTGGAA
*Ccl5*‐forward	GCTGCTTTGCCTACCTCTCC
*Ccl5*‐reverse	TCGAGTGACAAACACGACTGC

### 2.10 Stereotactic injection of adeno‐associated viruses (AAVs)

Briefly, the mice were deeply anesthetized with pentobarbital sodium and immobilized using a stereotactic device. A 0.5 mm burr hole was drilled into the skull. Vector particles were injected into the hippocampus (anteroposterior, −2.0 mm from bregma; mediolateral, 1.5 mm; dorsoventral, 2.0 mm) using a glass electrode at a rate of 0.2 μL/ min. When the injection was complete, the cannula was left to rest for 5 min to prevent efflux of the viral vector solution. The mice were allowed to recover for 2 weeks after surgery before the behavioral test. AAVs, including pAAV‐hSyn‐Ctss‐3xFlag‐P2A‐EGFP‐WPRE (titer: 1 × 10^12^ gc/μL), pAAV‐hSyn‐3xFlag‐P2A‐EGFP‐WPRE (titer: 1 × 10^12^ gc/μL), pAAV‐hSyn‐ EGFP‐3xFlag‐shRNA‐WPRE (titer: 1 × 10^12^ gc/μL) were constructed by OBIO Scientific Service.

### 2.11 Morris water maze (MWM)

Spatial learning and memory abilities of mice were tested by the MWM following a previous protocol with some modifications (Vorhees & Williams, 2006). Briefly, the mice were acclimatized to the test apparatus (120 cm in diameter) 1 day before the experiment. The maze filled with opacified water was replaced daily, and the water temperature was maintained at 19–22°C. The maze was artificially divided into four quadrants, and the platform (10 cm in diameter) was fixed 1 cm beneath the water surface and randomly placed in the center of one quadrant. Different colored and shaped objects were mounted on the quadrant walls as landmarks. During the training, the mice swam freely for 60 s to find the platform. The mice that failed to find the platform were guided to it and allowed to stay on it for 10 s. The mice were trained four times daily, and the data are the average of the four trials. The platform was removed 24 h after the last training trial, and the mice were tested for memory retention in a probe trial. The swimming activity of each mouse was monitored using a video camera mounted overhead and automatically recorded via EthoVision XT behavioral tracking software (Noldus, Wageningen, the Netherlands).

### 2.12 Open field test

All procedures were conducted in a square open‐field chamber (40.6 × 40.6 cm, Accuscan) mounted within sound‐attenuating shells. The behavior was monitored by a grid of invisible infrared light beams on arena walls. Data were collected and analyzed via EthoVision XT behavioral tracking software (Noldus, Wageningen, the Netherlands). The mice were exposed to the test chambers for 10 min to examine activity levels and habituation. Each mouse was placed at the center of the chamber and allowed to walk freely to start a session. The arena was cleaned with 70% ethanol after each mouse had completed a session.

### 2.13 RNA‐sequencing and data processing

The hippocampal tissues of five young wild‐type (WT) mice (2‐month‐old, male) and five aging WT mice (12‐month‐old, male) were collected, and total RNA was extracted using TRIzol. Besides, the hippocampal tissues of five control mice (WT, 2‐month‐old, male) and five CTSS‐overexpression mice (WT, 2‐month‐old) were collected, and total RNA was extracted. The RNA‐seq library was prepared and sequenced on the Illumina NovaSeq6000 platform with the PE150 strategy (Biolinker Technology Kunming Co., Ltd., Kunming, China). Read quality was assessed with FastQC, and reads were aligned to the mouse genome (mm10) using histat2 with default parameters. Aligned reads were subjected to Featurecount for read counting. Principal component analysis (PCA) and differential expression of genes between young and aging or WT and CTSS‐overexpression hippocampal tissues were all conducted in the R package. Gene Ontology (GO) categories were assessed and visualized with the R package clusterProfiler and enrichplot, respectively. Gene set enrichment analysis (GSEA) for the Kyoto Encyclopedia of Genes and Genomes (KEGG) pathways was performed on the rlog normalized counts from DESeq2 output with the criteria of |Log2FoldChange| >1 and FDR‐adjusted *p* <0.05.

### 2.14 Preparation of single‐cell suspensions

Single‐cell suspensions of hippocampal tissues were prepared to quantify the counts of immune cell subpopulations in the hippocampus using fluorescence‐activated cell sorting analysis (FACS). Young mice aged 2 months were deeply anesthetized and stereotactically injected with control and CTSS overexpression AAVs in the hippocampus. After 15 days, the mice were anesthetized, and the hippocampi were quickly removed after transcardial perfusion with cold PBS and then immersed in cold PBS. The brains were mechanically homogenized through 70‐μm cell strainers (BD Biosciences, Franklin Lake, New Jersey, USA) and centrifuged. To isolate hippocampal cells, 5 mL of 30% Percoll (GE Healthcare, Sweden, Chicago, Illinois, USA) was added to the cell pellets obtained from the brain tissues centrifuged at 700 × g for 10 min. After carefully aspirating the myelin layer and supernatant, the pellets were washed with PBS and reconstituted in FACS single‐cell suspensions.

### 2.15 FACS analysis

Fresh brain‐derived single‐cell suspensions at a density of 105–106 cells/100 μL in 1% BSA / PBS were incubated with mouse antigen‐specific antibodies conjugated to one type of fluorochrome, including PE, APC, APC/cy7, and BV421. Immune cell subsets in the brain were identified by extracellular staining with specific antibodies against the following antigens: CD11b (BioLegend, #101211), CD45.2 (BioLegend, #109823), CX3CR1 (BioLegend, #149005), and CD86 (BioLegend, #105031). For intracellular staining of CD206 (BioLegend, #141705), fixation and permeabilization were performed using a fixation/permeabilization kit (BioLegend, #426803). Finally, the samples were acquired on a FACS Aria III flow cytometer and analyzed with Flowjo software.

### 2.16 Transwell coculture system

To investigate the biological effects of microglia on CX3CL1 secretion from neurons in vitro, we cocultured BV2 and HT‐22 cells. Briefly, HT‐22 cells were seeded onto the upper chamber of a 12‐mm transwell culture plate (0.4‐μm pore size, polycarbonate membrane, Corning, New York, USA), and BV2 cells were plated onto the bottom chamber. Upon reaching confluence, HT‐22 cells were treated with pAAV‐hSyn‐3xFlag‐P2A‐EGFP‐WPRE and pAAV‐hSyn‐EGFP‐3xFlag‐Ctss‐WPRE viruses for 48 h. Afterward, the coculture media in the bottom chamber was collected using ELISA to evaluate CX3CL1 secretion. BV2 cells were collected to detect CX3CR1 protein levels using Immunoblot.

### 2.17 Transcripts per million (TPM) analysis of CTSS expression in database

We retrieved and obtained CTSS expression data in TPM from individuals with AD across different tissues (entorhinal cortex, hippocampus, temporal cortex, and frontal cortex) from the AlzData database (http://www.alzdata.org/). Corresponding TPM data from the same tissues in a healthy control population were collected for comparative analysis. Unpaired t test with Welch's correction was employed, and the results were visualized using box plots.

### 2.18 Cannula implantation and LY3000328 administration

Male *APP/PS1* mice aged 8 months were used for cannula implantation and LY3000328 (MedChemExpress, #HY‐15533, USA) administration. We implanted guide cannulas bilaterally above the hippocampus (anteroposterior, −2.0 mm from bregma; mediolateral, 1.5 mm; dorsoventral, 2.0 mm), allowing the animals to recover for 10 days before the following procedure. During the MWM test, we injected either dimethyl sulfoxide (DMSO) (*n* = 7) or 10 mM LY 3000328 (*n* = 6) per cannula with the concentration of 0.2 mg/kg at a rate of 0.2 μL/min for 5 consecutive days after the training trails of MWM test. After the test trials, we injected the same amount of DMSO (n = 7) or LY3000328 (n = 6) per cannula and performed the open field test the next day.

### 2.19 Quantification and statistical analysis

GraphPad Prism 6 (GraphPad, USA, La Jolla, California, USA) software was used to analyze all data. Comparisons between groups were made by unpaired or paired two‐tailed Student's t‐test, Mann–Whitney U test, χ^2^ test, one‐way ANOVA, or two‐way RM ANOVA. Statistical significance was represented as *p < 0.05; **p < 0.01; ***p < 0.001, and ****p < 0.0001. All data are presented as the mean ± standard errors of means (SEM).

## 3 RESULTS

### 3.1 CTSS expression is robustly upregulated in aging individuals and related to the aging‐related phenotypes

**FIGURE 1:**
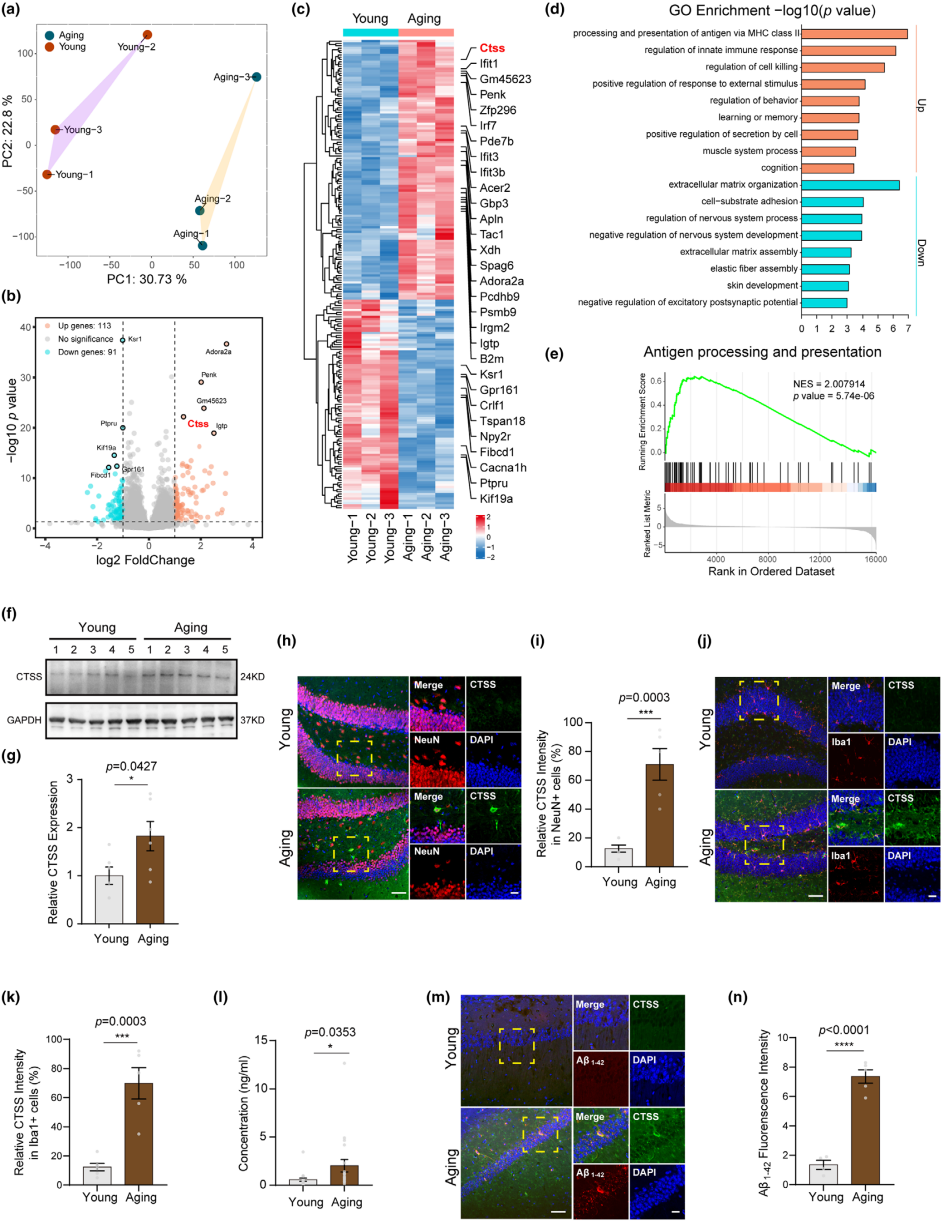
CTSS expression was upregulated in aging individuals and related to immune process and Aβ_1‐42_ accumulation. (a) PCA of RNA‐Seq on the brains of young and aging mice (*n* = 3 per group). (b) Volcano plots of RNA‐Seq demonstrating up‐and down‐regulated genes in aging mice using fold change and *p‐*value cutoffs of 1.0 and 0.05, respectively. (c) Heatmap displaying the top 30 DEGs and CTSS was included in the gene list. (d) GO enrichment analysis on DEGs identified the upregulated and downregulated processes. (e) DEGs were significantly enriched for the pathway of “antigen processing and presentation,” as established by GSEA analysis. (f) Immunoblot of CTSS in the two groups. (g) Immunoblot analysis of the protein levels of CTSS in other 12 mice in two groups (*n* = 6 per group). (h) IF showing that CTSS is colocalized with NeuN, and the IF intensity of CTSS in NeuN positive cells in aging mice was significantly higher than that in young mice. Scale bar: 20 μm. (i) Analysis of the relative CTSS intensity in NeuN positive cells in two groups (young: *N* = 6, aging: *N* = 5). (j) IF showing that CTSS is colocalized with Iba1, and the IF intensity of CTSS in Iba1 positive cells in aging mice was significantly higher than that in young mice. Scale bar: 20 μm. (k) Analysis of the relative CTSS intensity in Iba1 positive cells in two groups (young: *N* = 6, aging: *N* = 5). (l) ELISA assay showed that CTSS concentration in the serum of aging people is significantly higher than that in young people (*n* = 20 per group). (m) IF showed that the fluorescence intensity of Aβ_1‐42_ was significantly increased in the hippocampus of aging mice than that in the young mice, which was partially colocalized with CTSS. Scale bar: 20 μm. (n) Analysis of the Aβ_1‐42_ fluorescence intensity per slice (young: *N* = 4, aging: *N* = 5). T‐test was used for (g, i, k, l, and n) Data are expressed as mean ± SEM. *p < 0.05.

To screen the genes that play vital roles in the brains of aging mice, we performed the RNA sequencing in the hippocampus of young and aging WT male mice (Table S1). PCA revealed substantial differences in transcriptional patterns between young and aging mice (Figure 1a). Subsequently, we used DESeq2 to identify differentially expressed genes (DEGs) in aging and young brains. Using the criteria of |Log2FoldChange| >1 and *p*‐value <0.05, we identified 204 DEGs in aging mice compared to the young mice, with 113 upregulated and 91 downregulated genes (Figure 1b). The top 30 DEGs with the greatest changes in expression are displayed in Figure 1c. Among the DEGs, CTSS, a member of cysteine protease cathepsin family, was significantly upregulated in the aging group (Figure 1b,c). Gene Ontology (GO) enrichment analysis of DEGs identified upregulated processes involved in antigen processing and presentation of peptide antigens via MHC class II, innate immune response regulation, learning and memory, recognition, and so on. While the downregulated processes included the extracellular matrix organization, cell‐substrate adhesion, nervous system regulation, and so on (Figure 1d). Next, we applied Gene set enrichment analysis (GSEA) to identify the pathways that were differentially expressed between aging and young mice. Consistently, GSEA demonstrated significant enrichment (*p* < 0.05) in antigen processing and presentation in aging mice (Figure 1e). These results revealed that CTSS expression was significantly upregulated in the hippocampus of aging mice and highlighted its potential relevance in immune processes associated with aging.

We subsequently performed the immunoblot assay and immunofluorescence (IF) assay to verify the result of RNA Seq. We found the CTSS protein levels were significantly increased in the hippocampus of aging mice **(**Figure 1f,g **)**. Additionally, we observed a markedly elevated fluorescence intensity of CTSS both in the neurons and microglia of aging mice compared to the young ones (Figure 1h–k), which suggested that the increased expression of CTSS in the hippocampus of the aging mice is attributable to both the neuronal and microglia sources. Previous studies shown that CTSS is only expressed in microglia. Our study revealed for the first time that CTSS is also expressed in neurons and positively correlated with age. Besides, we also observed an elevated fluorescence intensity both in the neurons and microglia in the prefrontal cortex of aging mice compared to the young ones (Figure S1a–d). Subsequently, to investigate whether CTSS expression is also age‐related in human populations, we measured CTSS concentration in the serum of young and elderly individuals using ELISA. Consistent with the findings from the mice experiments, the serum levels of CTSS were significantly higher in the elderly than in the younger individuals (Figure 1l). Consequently, our results indicated that CTSS expression is robustly upregulated in aging individuals.

Age constitutes the predominant risk factor for numerous neurodegenerative disorders, including AD (Hou et al., 2019). To explore the potential correlation between the CTSS expression and AD symptoms, we employed the young (2‐month‐old, male) and aging (12‐month‐old, male) WT male mice, and costained CTSS alone with beta Amyloid 1–42 (Aβ_1‐42_) to explore the potential correlation between CTSS and AD symptoms in the hippocampus. Notably, we observed that the fluorescence intensity of Aβ_1‐42_ was signifcantly increased in aging mice. Besides, CTSS was partially colocalized with the Aβ_1‐42_, which suggested that CTSS might be involved in the transition or clearance of Aβ_1‐42_ (Figure 1m,n). Overall, our data revealed a positive correlation between CTSS expression, age, and age‐related phenotypes.

### 3.2 CTSS overexpression in hippocampus neurons impaired the spatial learning and memory behavior in young mice

**FIGURE 2:**
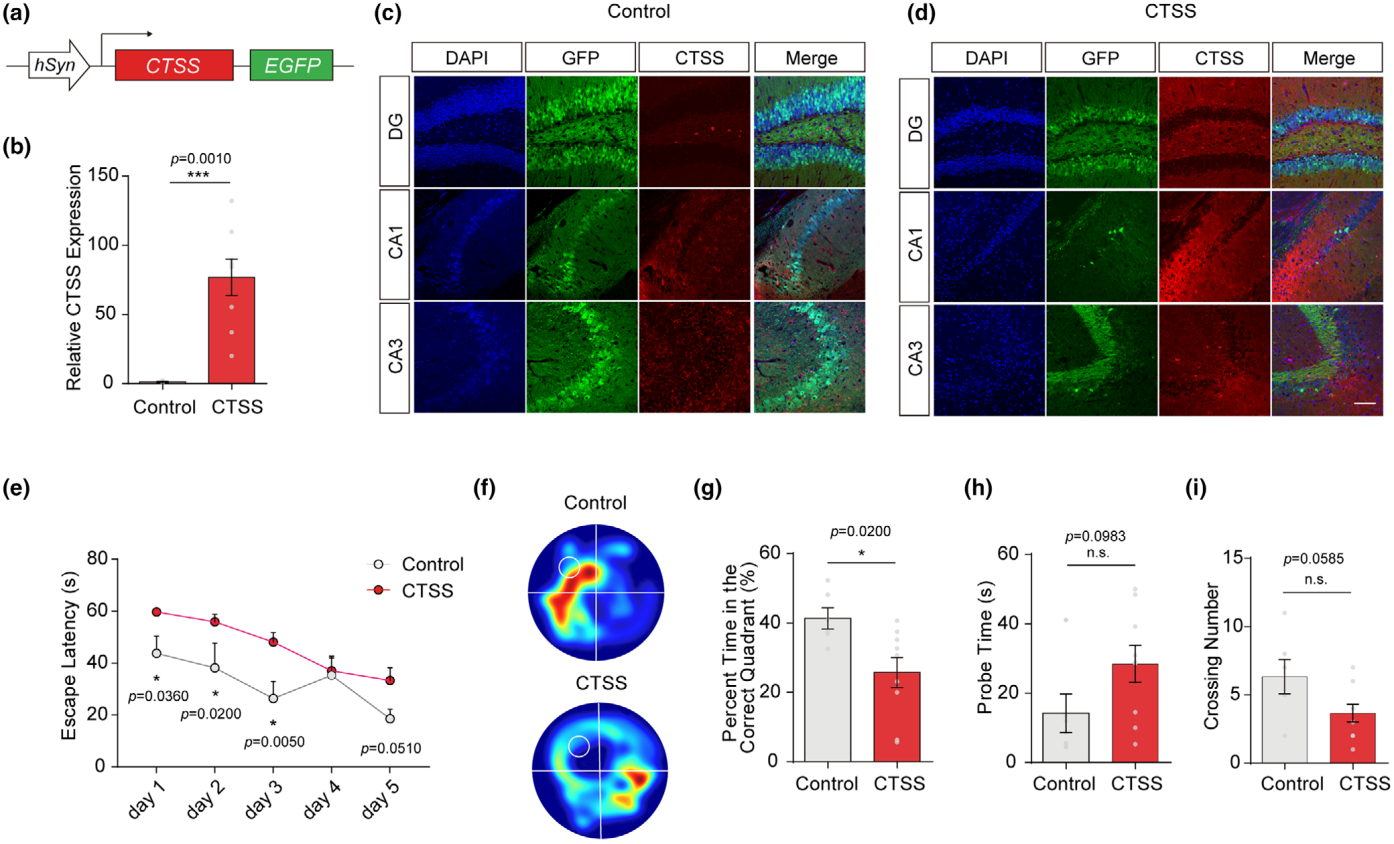
CTSS overexpression in hippocampus neurons impaired the spatial learning and memory behavior in young mice. (a) Schematic representation of AAV‐hSyn‐Ctss‐3xFlag‐P2A‐EGFP‐WPRE construct. (b) Quantitative RT‐PCR analysis of the gene expression level of CTSS in two groups (control: *N* = 5, CTSS: *N* = 8). (c, d) IF images injected with AAV‐hSyn‐Ctss‐3xFlag‐P2A‐EGFP‐WPRE reveal that CTSS expression is increased in multiple subregions of the hippocampus, including the CA1, CA3, and DG. Scale bar: 20 μm. (e) Escape latencies measured as meantime (s) during five consecutive training days. (f) Heat plots of search intensity during probe trials conducted on day 6. A high dwell time across the MWM pool area is indicated by colors close to red, whereas colors close to blue indicate a lower dwell time. (g) The percentage of time spent in the target quadrant during the probe trials on day 6 (control: *N* = 6, CTSS: *N* = 9). (h) The latency of the first target‐site crossover (probe time) during the probe trials on day 6 (control: *N* = 6, CTSS: *N* = 9). (i) The average crossing number over the platform site during the probe trials on day 6 (control: *N* = 6, CTSS: *N* = 9). Two‐way RM ANOVA test was used for (e) T‐test was used for (b, g–i). Data are presented as mean ± SEM. *: *p* < 0.05, **: *p* < 0.01, ***: *p* < 0.001.

To verify the role of neuronal CTSS, we overexpressed CTSS in young mice aged 2‐month‐old via injecting an AAV (Figure 2a). After 15 days' recovery, we found that the virus was highly expressed in multiple subregions of the hippocampus, including the CA1, CA3, and dental gyrus (DG) regions, and the CTSS fluorescence intensity was significantly increased in the overexpression group (Figure 2c,d). Besides, we found a significant increase in the CTSS mRNA expression level in the hippocampus of the overexpressed group (Figure 2b).

Spatial learning and memory abilities were evaluated using MWM tests. The results indicated that CTSS overexpression in hippocampus neurons significantly impaired the spatial learning abilities, as shown by the longer escape latencies of mice in the overexpressed group during five consecutive training days (Figure 2e). Moreover, CTSS overexpression in the hippocampus impaired the spatial memory of young mice, as shown by less time in the target quadrant (Figure 2f,g), more time for the first target‐site crossover (probe time) in the platform area (Figure 2h), and decreased crossing number (Figure 2i) of the overexpressed group compared to the control group. These findings collectively demonstrated that CTSS overexpression in hippocampus neurons induced spatial learning and memory deficits in young mice. The results of the open field test indicated comparable levels of exploratory activity between the overexpression and control groups (Figure S2a–c). Additionally, the overexpressed group did not exhibit anxiety‐like behaviors, as evidenced by an equal time spent in centers compared to the control group (Figure S2d). These results suggested that the overexpression group's impaired learning and memory phenotypes were not attributable to altered motor ability.

### 3.3 CTSS knockdown in hippocampus neurons rescued the spatial learning and memory deficits in aging mice

**FIGURE 3:**
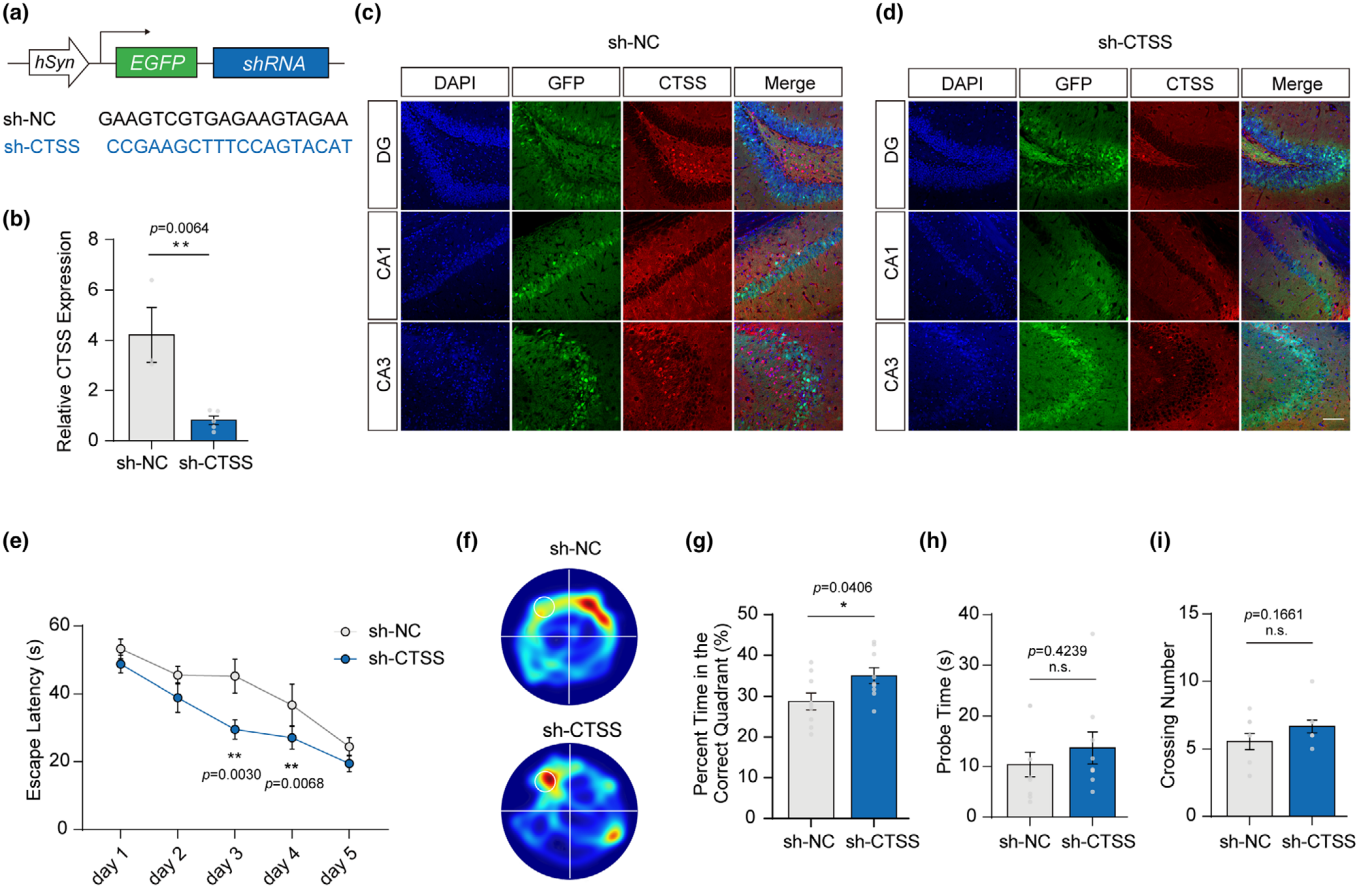
CTSS knockdown in hippocampus neurons rescued the spatial learning and memory deficits in aging mice. (a) Schematic representation of AAV‐hSyn‐ EGFP‐3xFlag‐shRNA‐WPRE construct. (b) Quantitative RT‐PCR analysis of the gene expression level of CTSS in two groups (sh‐NC: *N* = 3, sh‐CTSS: *N* = 5). (c, d) IF images injected with AAV‐hSyn‐Ctss‐3xFlag‐P2A‐EGFP‐WPRE reveal that CTSS expression is reduced in multiple subregions of the hippocampus, including the CA1, CA3, and DG. Scale bar: 20 μm. (e) Escape latencies measured as meantime (s) during five consecutive training days. (sh‐NC: *N* = 9, sh‐CTSS: *N* = 9). (f) Heat plots of search intensity during probe trials conducted on day 6. (g) The percentage of time spent in the target quadrant during the probe trials on day 6 (sh‐NC: *N* = 9, sh‐CTSS: *N* = 9). (h) The latency of the first target‐site crossover (probe time) during the probe trials on day 6 (sh‐NC: *N* = 9, sh‐CTSS: *N* = 9). (i) The average crossing number over the platform site during the probe trials on day 6 (sh‐NC: *N* = 9, sh‐CTSS: *N* = 9). Two‐way RM ANOVA test was used for (e) T‐test was used for (b, g–i) Data are presented as mean ± SEM. *: *p* < 0.05, **: *p* < 0.01, ***: *p* < 0.001.

Subsequently, CTSS was depleted in hippocampus neurons of WT aging mice (12‐month‐old, male) by injecting AAVs, and learning and memory abilities were evaluated (Figure 3a). After 15 days' recovery, we found that the virus was highly expressed in multiple subregions of the hippocampus, including the CA1, CA3, and the DG regions, and the CTSS fluorescence intensity was significantly decreased in the CTSS ablation group (Figure 3c,d). Besides, we found a significant decrease in the CTSS mRNA expression level in the hippocampus of the CTSS depletion group (Figure 3b).

In the MWM test, the mice in the CTSS knockdown group displayed significantly improved learning ability compared to the negative control (NC) group, as shown by a shorter escape latency to the platform during five consecutive training days (Figure 3e). Moreover, mice in the CTSS depletion group spent more time in the target quadrant during the probe trials (Figure 3f,g), had significantly shorter latency for the first entry to the target (platform) (Figure 3h), and more target entries (Figure 3i) compared to the NC group, indicating better memory retention of the mice in the CTSS depletion group. Collectively, these data demonstrated that CTSS knockdown rescued the spatial learning and memory deficits in aging mice. The results of open field test suggested that the comparable activity of the mice in the CTSS depletion group was comparable to that of the NC group (Figure S3a), as revealed by an equal level of the total distance traveled and the average velocity (Figure S3b,c). Moreover, the mice in the CTSS knockdown group did not exhibit anxiety‐like behaviors, reflected by the equal time spent in centers compared to those in the control group (Figure S3d). These results indicated that alleviating these phenotypes in the CTSS depletion group did not result from the altered motor ability.

### 3.4 Neuronal CTSS overexpression aggravated brain inflammatory milieu

**FIGURE 4:**
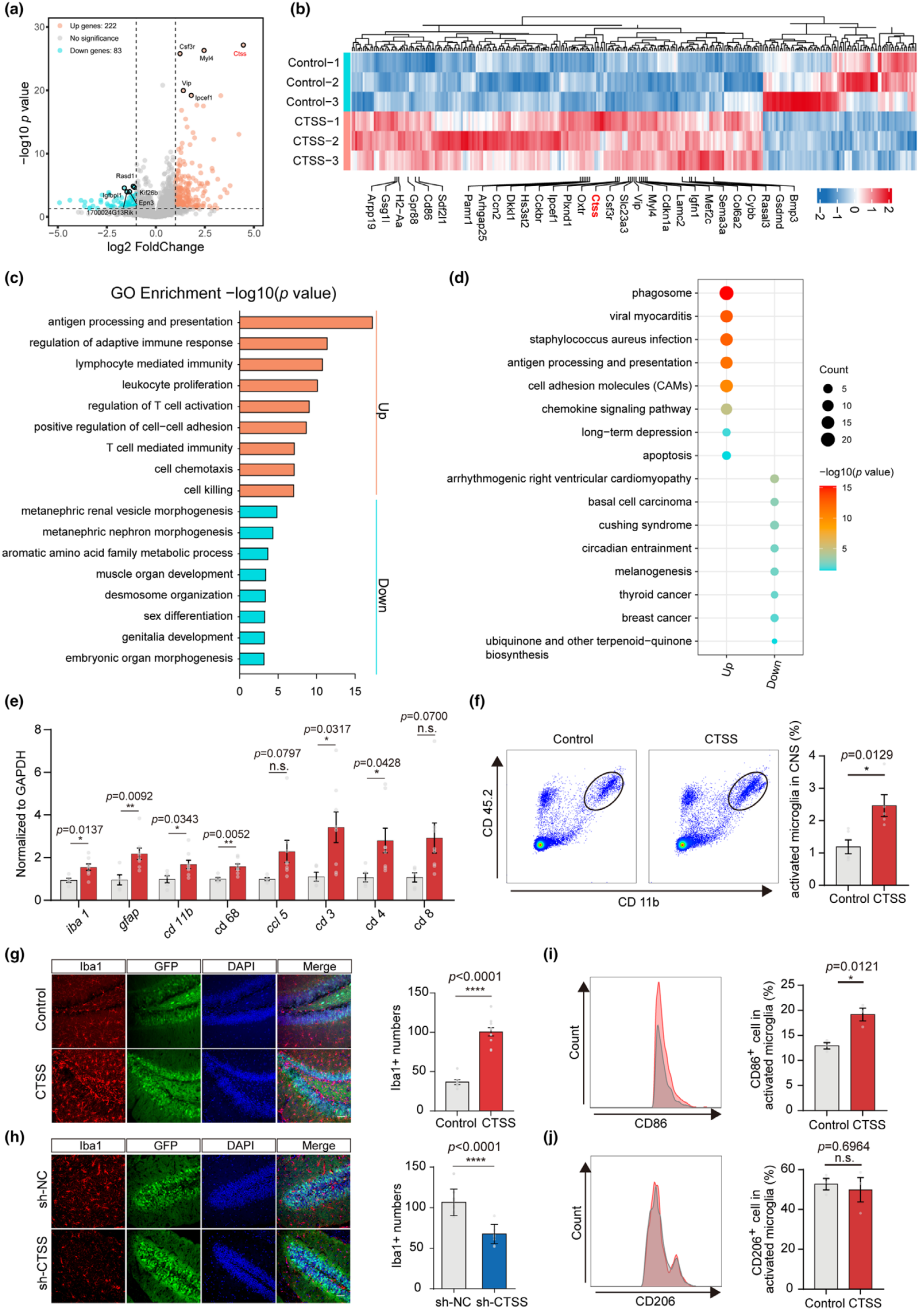
CTSS overexpression in hippocampus neurons leads to inflammatory milieu with microglia activation to M1 phenotype. (a) Volcano plots of RNA‐Seq displaying up‐and down‐regulated genes in CTSS overexpressed mice using fold change and *p*‐value cutoffs of 1 and 0.05, respectively (*n* = 3 per group). (b) Heatmap displaying the top 30 DEGs in CTSS overexpression mice and CTSS was included in the gene list. (c) GO enrichment analysis on DEGs identified the upregulated processes and the down‐regulated processes in CTSS overexpression mice. (d) KEGG pathway enrichment analysis of DEGs in CTSS overexpression mice. (e) Quantitative RT‐PCR analysis of the expression level of multiple DEGs responsible for neuroinflammation pathway in two groups (Control: *N* = 5, CTSS: *N* = 8). (f) FACS analysis of the CD11b^+^ CD45^int^ microglia in two groups (*n* = 5 per group). (g) (left) Immunostaining of Iba1 (red), GFP (green), and DAPI (blue) in the hippocampus of mice injected with control and CTSS overexpressed virus. Scale bar: 20 μm; (right) quantification of Iba1 numbers in two groups (Control: *N* = 7, CTSS: *N* = 10). (h) (left) Immunostaining of Iba1 (red), GFP (green), and DAPI (blue) in the hippocampus of mice injected with negative control (sh‐NC) and CTSS knockdown (sh‐CTSS) virus. Scale bar: 20 μm; (right) quantification of Iba1 numbers in two groups (*n* = 9 per group). (i) FACS analysis of the expression of CD86 in CD11b^+^ CD45^int^ microglia in two groups (*n* = 3 per group). (j) FACS analysis of the expression of CD206 in CD11b^+^ CD45^int^ in two groups (*n* = 3 per group). T‐test was used for (e–j) Data are presented as mean ± SEM. *: *p* < 0.05, **: *p* < 0.01, ***: *p* < 0.001.

To further explore potential mechanisms underlying the function of CTSS on spatial learning and memory, we performed RNA sequencing of the CTSS overexpressed hippocampus and identified 305 DEGs compared to the control, including 222 upregulated and 83 downregulated genes (|log2FoldChange| >1; *p*‐value <0.05), and CTSS was identified as the most significantly upregulated DEG (Table S2) (Figure 4a,b). GO enrichment analyses unveiled that the upregulated genes were predominantly involved in immune‐related pathways. while, the downregulated genes were enriched in pathways related to kidney development, amino acid metabolism, and embryonic development (Figure 4c). The Kyoto Encyclopedia of Genes and Genomes (KEGG) analysis highlighted enrichment in pathways like phagosome, viral myocarditis, *Staphylococcus aureus* infection, antigen processing, and presentation in the CTSS overexpression group (Figure 4d). These results indicated a potential role of CTSS in neuroinflammation pathway. To further validate these findings, we performed the quantitative reverse transcription polymerase chain reaction (qRT‐PCR) and compared the expression of DEGs associated with the neuroinflammation pathway in the two groups. Consistently, the mRNA expression levels of seven inflammatory markers, including *Gfap*, *Iba1*, *CD11b*, *CD68*, *CD3*, and *CD4*, were significantly elevated in the overexpression group. Besides, the expression level of the other three genes, including the *Ccl5* and *Cd8a*, were also upregulated in the overexpression group **(**Figure 4e **)**.

In summary, our data suggested that elevated CTSS expression levels may impact the neuroinflammation pathway, potentially contributing to impaired spatial learning and memory.

### 3.5 Neuronal CTSS overexpression exhibited pro‐inflammatory properties with microglia activation to M1 phenotype

It is known that activated microglia orchestrate both initiation and progression of inflammatory responses (Skaper et al., 2017). We therefore assessed the microglia activation degree after CTSS overexpression in hippocampus neurons of young mice. We observed that CTSS overexpression significantly increased the number of microglia in the hippocampal using FACS analysis gating on CD11b^+^ CD45^int^ microglia (Figure 4f). Moreover, immunofluorescent staining revealed a significantly increase in microglia proliferation in CTSS overexpression group. Remarkably, CTSS overexpression induced a notable transformation in microglial morphology, shifting from ramified to amoeboid (Figure 4g). Consistently, CTSS knockdown in hippocampus neurons of aging mice significantly reduced the number of Iba1‐positive cells and was accompanied by a transformation in microglial morphology from amoeboid to ramified (Figure 4h).

Microglial phenotypes are associated with effector function, and the transition from the M1 phenotype to the M2 phenotype reflects the resolution of inflammation (Paolicelli et al., 2022). To investigate the effect of CTSS overexpression on microglial phenotypes, we used FACS analysis gating on CD11b^+^ CD45^int^ microglia. Despite a comparable proportion of CD206^+^ anti‐inflammatory microglia, a larger proportion of primed microglia expressing pro‐inflammatory marker CD86 was observed in the CTSS overexpression group (Figure 4i,j). The results indicated that neuronal CTSS overexpression could effectively promote the microglia activation to M1 pro‐inflammatory phenotype.

Taken together, our findings suggested that CTSS overexpression in neurons leads to microglial activation and polarization towards a M1 pro‐inflammatory phenotype, thereby aggravating neuroinflammation and disease outcomes.

### 3.6 Neuronal CTSS modulated neuroinflammation and cognition behavior via CX3CL1‐CX3CR1 axis and JAK2‐STAT3 signaling pathway

**FIGURE 5:**
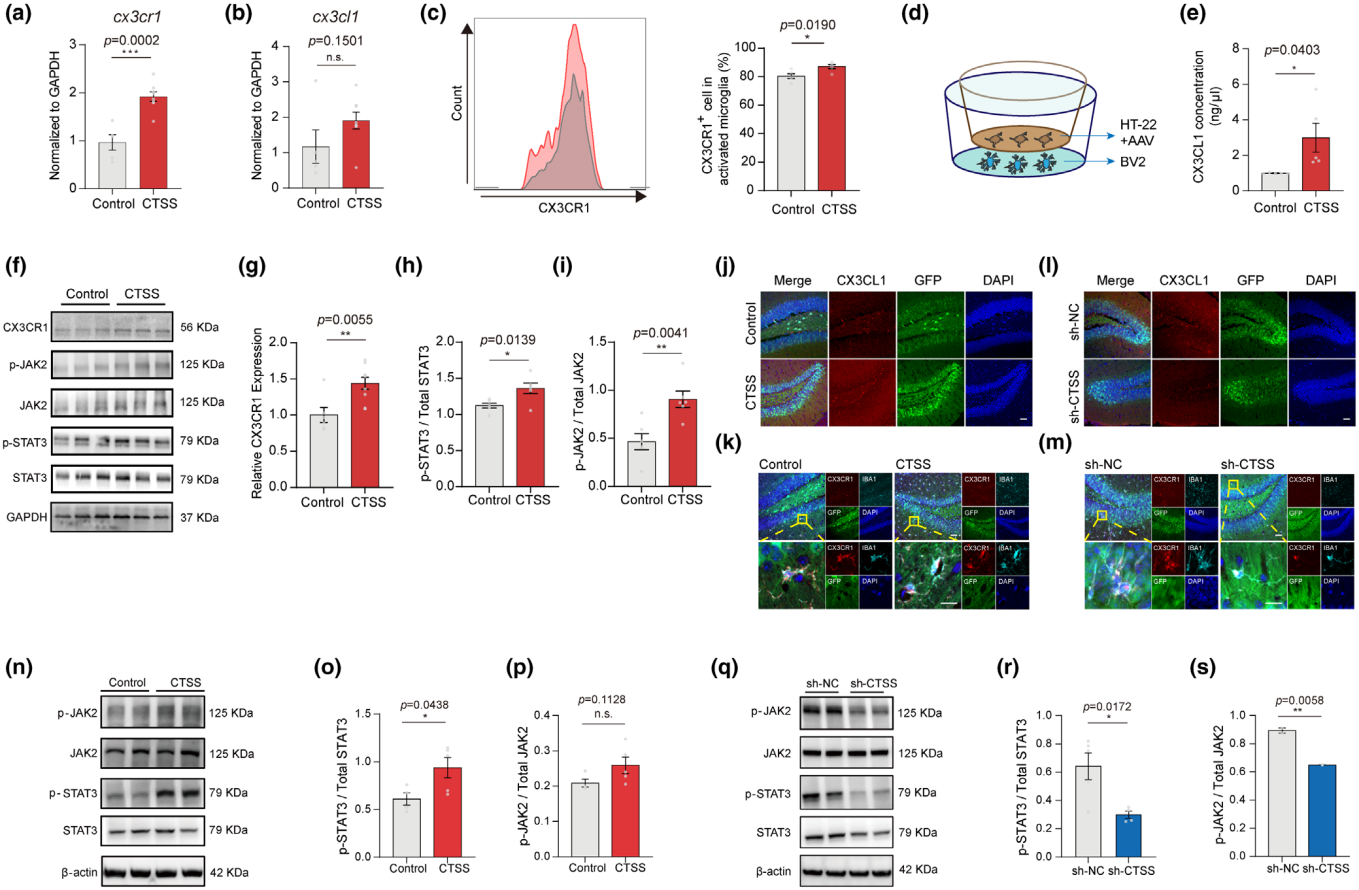
CTSS modulates neuroinflammation and cognition behavior via the CX3CL1‐CX3CR1 axis and JAK2‐STAT3 signaling pathway. (a) Quantitative RT‐PCR analysis of the expression level of cx3cr1 in two groups (Control: *N* = 5, CTSS: *N* = 8). (b) Quantitative RT‐PCR analysis of the expression level of cx3cl1 in two groups (Control: *N* = 5, CTSS: *N* = 8). (c) FACS analysis of the expression of CX3CR1 in CD11b^+^ CD45^int^ microglia in two groups (*n* = 5 per group). (d) Illustration of the in vitro coculture system. (e) ELISA assay showed that CX3CL1 concentration in the medium of CTSS group is significantly higher than that in control group (*n* = 5 per group). (f) Immunoblot of CX3CR1 and JAK2‐STAT3 pathway in two groups. (g) Immunoblot analysis of the protein levels of CX3CR1 in two groups (control: *N* = 6, CTSS: *N* = 9). (h, i) Immunoblot analysis of JAK2‐STAT3 pathway in control and CTSS overexpressed groups (Control: *N* = 6, CTSS: *N* = 6). (j) Immunostaining of CX3CL1 (red), GFP (green), and DAPI (blue) in the DG subregions in control and CTSS overexpressed groups. Scale bar: 20 μm. (K) Immunostaining of CX3CR1 (red), Iba1 (cyan), GFP (green), and DAPI (blue) in the DG subregions in control and CTSS overexpressed groups. Scale bar: 20 μm. (l) Immunostaining of CX3CL1 (red), GFP (green), and DAPI (blue) in the DG, CA1, and CA3 subregions in NC and CTSS knockdown groups. Scale bar: 20 μm. (m) Immunostaining of CX3CR1 (red), Iba1 (cyan), GFP (green), and DAPI (blue) in the DG, CA1, and CA3 subregions in NC and CTSS knockdown groups. Scale bar: 20 μm. (n‐p) Immunoblot analysis of JAK2‐STAT3 pathway in control and CTSS overexpressed groups (Control: *N* = 4, CTSS: *N* = 5). (q–s) Immunoblot analysis of JAK2‐STAT3 pathway in NC and CTSS knockdown groups (sh‐NC: *N* = 5, sh‐CTSS: *N* = 4). T‐test was used for (a–c, e, g–i, o, p, r, and s) Data are expressed as mean ± SEM, *: *p* < 0.05, **: *p* < 0.01, ***: *p* < 0.001.

Studies have revealed that the CX3CL1‐CX3CR1 axis is closely related to neuroinflammation by regulating neuronal activity that controls microglial functions (Subbarayan et al., 2022). Based on this, we initially investigated the impact of neuron CTSS overexpression on the CX3CL1‐CX3CR1 axis. We observed elevated CX3CR1 and CX3CL1 mRNA levels via qRT‐PCR (Figure 5a,b). Consistently, FACS analysis revealed that CTSS overexpression significantly increased the number of CX3CR1‐positive cells (Figure 5c). Since CX3CL1 is secreted by neurons and interacts with CX3CR1 receptors in microglia (Zhang, Zhang, et al., 2023), we examined whether CTSS overexpression in neurons was sufficient to increase CX3CR1 expression in microglia. HT‐22 cells were treated with control or CTSS overexpression AAVs and cocultured with BV2 cells (Figure 5d). Following 48 h treatment, we detected higher CX3CL1 concentrations in the coculture media of the overexpression group than that of the control group (Figure 5e). CX3CR1 protein levels in BV2 cells were also significantly increased in the overexpression group (Figure 5f,g). We also cocultured primary neurons with microglia (Figure S4A), and observed that treatment of primary neurons with CTSS overexpression AAV did not significantly increase the concentration of CX3CL1 in the medium (Figure S4b). However, it markedly increased the CX3CR1 protein level in microglia (Figure S4c,d). Following that, we detected the CX3CL1 and CX3CR1 levels in vivo. IF staining revealed a significant elevation in the fluorescence intensity of CX3CL1 and CX3CR1 in multiple subregions of the hippocampus after neuron CTSS overexpression, including the DG (Figure 5j,k), CA1, and CA3 (Figure S5a,b). We had observed a clear colocalization of CX3CR1 with the microglia marker Iba1 in the DG, CA1, and CA3 subregions of the hippocampus. Notably, the neuronal CTSS overexpression induced a morphological transition of CX3CR1 from a ramified to an amoeboid form in young mice, which was consistent with the alterations in the morphology of microglial. In summary, our findings indicated that the elevated CTSS expression in neurons promotes the expression of the CX3CL1‐CX3CR1 axis, which plays the significant role in neuron–microglia “crosstalk.”

Subsequently, we examined the downstream CX3CL1‐CX3CR1 axis through immunoblot and found substantial elevation in the protein levels of p‐JAK2 and p‐STAT3 due to neuron CTSS overexpression in the hippocampus, indicating enhanced activation of the JAK2‐STAT3 pathway (Figure 5n–p). Besides, we also detected the JAK2‐STAT3 pathway in the BV2 cells in the coculture system shown in Figure 5d, and found that the JAK2‐STAT3 pathway were activated due to CTSS overexpression in HT‐22 cells (Figure 5f,h,i). Additionally, we assessed the p‐CREB expression level in the hippocampus through IF and observed no difference between the two groups. This result suggested that CTSS does not exert its function through the downstream CREB pathway (Figure S6a–d).

Conversely, CTSS knockdown in hippocampus neurons of aging mice significantly reduced the fluorescence intensity of CX3CL1 and CX3CR1 in multiple subregions, including DG (Figure 5l,m), CA1, and CA3 (Figure S5c,d). Neuronal CTSS knockdown induced a morphological transition of CX3CR1 from amoeboid to ramified in aging mice, which was consistent with the alterations in the morphology of microglial. Meanwhile, the protein levels of p‐JAK2 and p‐STAT3 were downregulated, suggesting that the JAK2‐STAT3 signaling pathway was inhibited by CTSS knockdown (Figure 5q–s).

Taken together, our data suggest that CTSS is vital in modulating neuroinflammation and cognitive behavior through the CX3CL1‐CX3CR1 axis and JAK2‐STAT3 pathway. Furthermore, our results underscored the involvement of neuron CTSS in intricate neuron–microglia “crosstalk” for the first time.

### 3.7 CTSS inhibition by LY3000328 rescued the AD‐related pathological features in APP/PS1 mice

**FIGURE 6:**
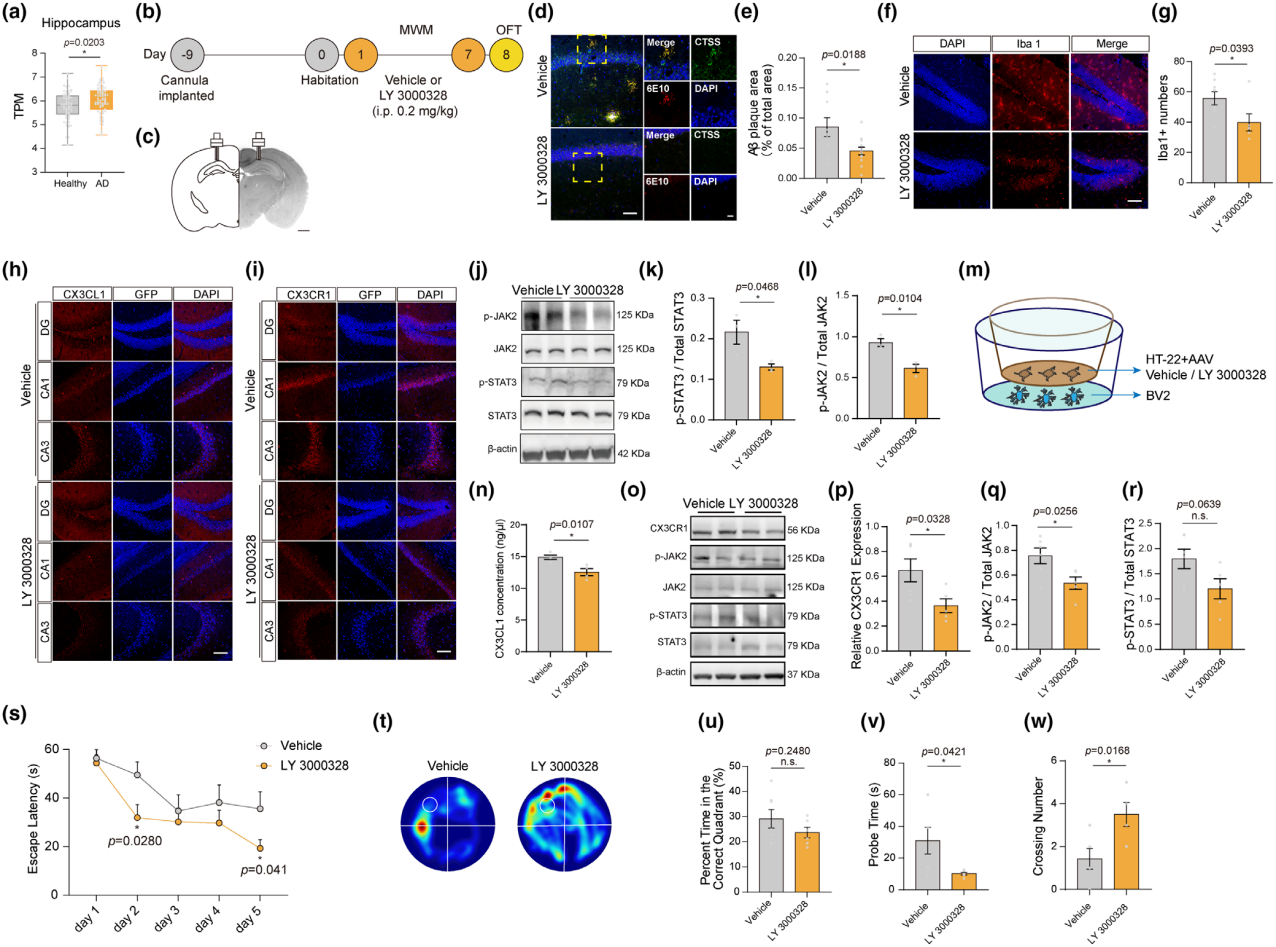
CTSS Inhibition by LY3000328 rescued AD‐related pathological features in *APP/PS1* mice. (a) The CTSS Transcripts Per Million (TPM) in the hippocampus of AD patients was significantly higher than those in healthy controls (Healthy: *N* = 66, AD: *N* = 74). (b) Time schedule of the experiments. (c) Coronal section depicting cannula implantation in the hippocampus. Scale bar: 1 mm. (d) Immunostaining of 6E10 (red), CTSS (green) and DAPI (blue) in the hippocampus of mice in two groups. Scale bar: 20 μm. (e) Quantification of the Aβ plaque area in two groups (vehicle: *N* = 9, LY3000328: *N* = 12). (f) Immunostaining of Iba1 (red) and DAPI (blue) in the hippocampus of mice in two groups. Scale bar: 20 μm. (g) Quantification of Iba1 numbers in the hippocampus of mice in two groups (vehicle: *N* = 8, LY3000328: *N* = 6). (h) Immunostaining of CX3CL1 (red) and DAPI (blue) in the DG, CA1, and CA3 subregions in two groups. Scale bar: 20 μm. (i) Immunostaining of CX3CR1 (red) and DAPI (blue) in the DG, CA1, and CA3 subregions in two groups. Scale bar: 20 μm. (j–l) Immunoblot analysis of JAK2‐STAT3 pathway in two groups (*n* = 3 per group). (m) Illustration of the in vitro coculture system. (n) ELISA assay showed that CX3CL1 concentration in the medium of LY 3000328 group is significantly lower than that in Vehicle group (n = 5 per group). (o) Immunoblot of CX3CR1 and JAK2‐STAT3 pathway in two groups. (p) Immunoblot analysis of the protein levels of CX3CR1 in two groups (Vehicle: *N* = 6, LY 3000328: *N* = 5). (q, r) Immunoblot analysis of JAK2‐STAT3 pathway in control and CTSS overexpressed groups (Vehicle: *N* = 6, LY 3000328: *N* = 5). (s) Escape latencies measured as meantime (s) during five consecutive training days. (*n* = 7 per group). (t) Heat plots of search intensity during probe trials conducted on day 6. High dwell time across the MWM pool area is indicated by colors close to red, whereas colors close to blue indicate lower dwell time. (u) The percentage of time spent in the target quadrant during the probe trials on day 6 (vehicle: *N* = 7, LY3000328: *N* = 6). (v) The latency of the first target‐site crossover (probe time) during the probe trials on day 6 (vehicle: *N* = 7, LY3000328: *N* = 6). (w) The average crossing number over the platform site during the probe trials on day 6 (vehicle: *N* = 7, LY3000328: *N* = 6). A two‐way RM ANOVA test was used for (s) T‐test was used for (a, e, g, k, l, n, p–r, u–w) Data are presented as mean ± SEM. *: *p* < 0.05, **: *p* < 0.01, ***: *p* < 0.001.

To investigate the association between CTSS and AD, we initially retrieved and obtained CTSS expression data from AD patients across various brain regions (entorhinal cortex, hippocampus, temporal cortex, and frontal cortex) from the AlzData database (http://www.alzdata.org/). Comparing with their healthy controls, we found a significant increase in CTSS TPM in the hippocampus of AD patients (Figure 6a). Furthermore, we observed a notable elevation of CTSS TPM in the temporal cortex of the AD patients (Figure S7a). Although the CTSS TPM levels in the entorhinal cortex and frontal cortex of AD patients were higher than those of their healthy counterparts, the differences were not statistically significant (Figure S7b,c). To further elucidate the role of CTSS in the pathogenesis of AD, we used *APP/PS1* transgenic mice as the AD model mice and reduced CTSS expression through stereotactic injection of the CTSS selective inhibitor LY3000328 using an implanted cannula in the hippocampus. Eight‐month‐old male *APP/PS1* mice were divided into vehicle and LY3000328 groups. The experiment schedule was shown in Figure 6b,c. The IF analysis revealed that LY3000328 treatment significantly reduced the CTSS level, together with a substantial decrease in Aβ deposition in the hippocampus of *APP/PS1* mice (Figure 6d,e). The Iba1‐positive cells in the hippocampus of *APP/PS1* transgenic mice were also significantly decreased in the LY3000328 group (Figure 6f,g). Subsequent IF analysis revealed that LY3000328 treatment significantly reduced the fluorescence intensity of CX3CL1 and CX3CR1 in multiple hippocampal subregions, including the CA1, CA3, and DG (Figure 6h,i). Immunoblot analysis indicated that LY 3000328 treatment significantly decreased p‐JAK2 and p‐STAT3 protein levels, suggesting that LY 3000328 exerts its beneficial role by inhibiting the JAK2‐STAT3 signaling pathway (Figure 6j–l). The effect of LY 3000328 treatment on neuron–microglia “crosstalk” were detected using the coculture system at the same time (Figure 6m). The result revealed that LY 3000328 significantly decreased the elevated CX3CL1 concentrations due to the CTSS overexpression in HT‐22 cells (Figure 6n). The protein levels of CX3CR1, p‐JAK2, and p‐STAT3 in BV2 cells were also reduced due to the LY 3000328 treatments (Figure 6o–r), indicating LY 3000328 acts to the CX3CL1‐CX3CR1 axis and JAK2‐STAT3 pathway in neuron–microglia “crosstalk.”

Finally, we investigated whether CTSS inhibition using LY 3000328 could rescued recognition deficient in *APP/PS1* mice. In the MWM test, LY 3000328 treatment in the hippocampus significantly improved learning and memory behavior, as shown by the shorter escape latency to the platform during the training trials **(**Figure 6s **)**, shorter latency for the first entry to the target (platform), more target entries, and increased time spent in the target quadrant during the probe trial (Figure 6u–w). The spatial distribution of the exploratory activity in the MWM probe trial is presented in Figure 6t. Following the probe trial of the MWM test, we administered the same amount of either DMSO or LY3000328 and performed the open field test the following day. The open field results revealed that the LY 3000328 injection did not affect the activity level of mice (Figure S8A–C). Moreover, LY3000328 injection in the hippocampus did not induce anxiety‐like behavior compared to vehicle in *APP/PS1* transgenic mice (Figure S8D).

In summary, our data revealed that LY3000328 could alleviate the neuroinflammation response, decrease Aβ deposition, and rescue the learning and memory deficits in *APP/PS1* mice via the CX3CL1‐CX3CR1 axis and JAK2‐STAT3 pathway, suggesting that CTSS has great potential for use as an effective target for treating AD.

### 3.8 Neuronal CTSS overexpression increased CTSB protein level, but decreased CTSL protein level in microglia

**FIGURE 7:**
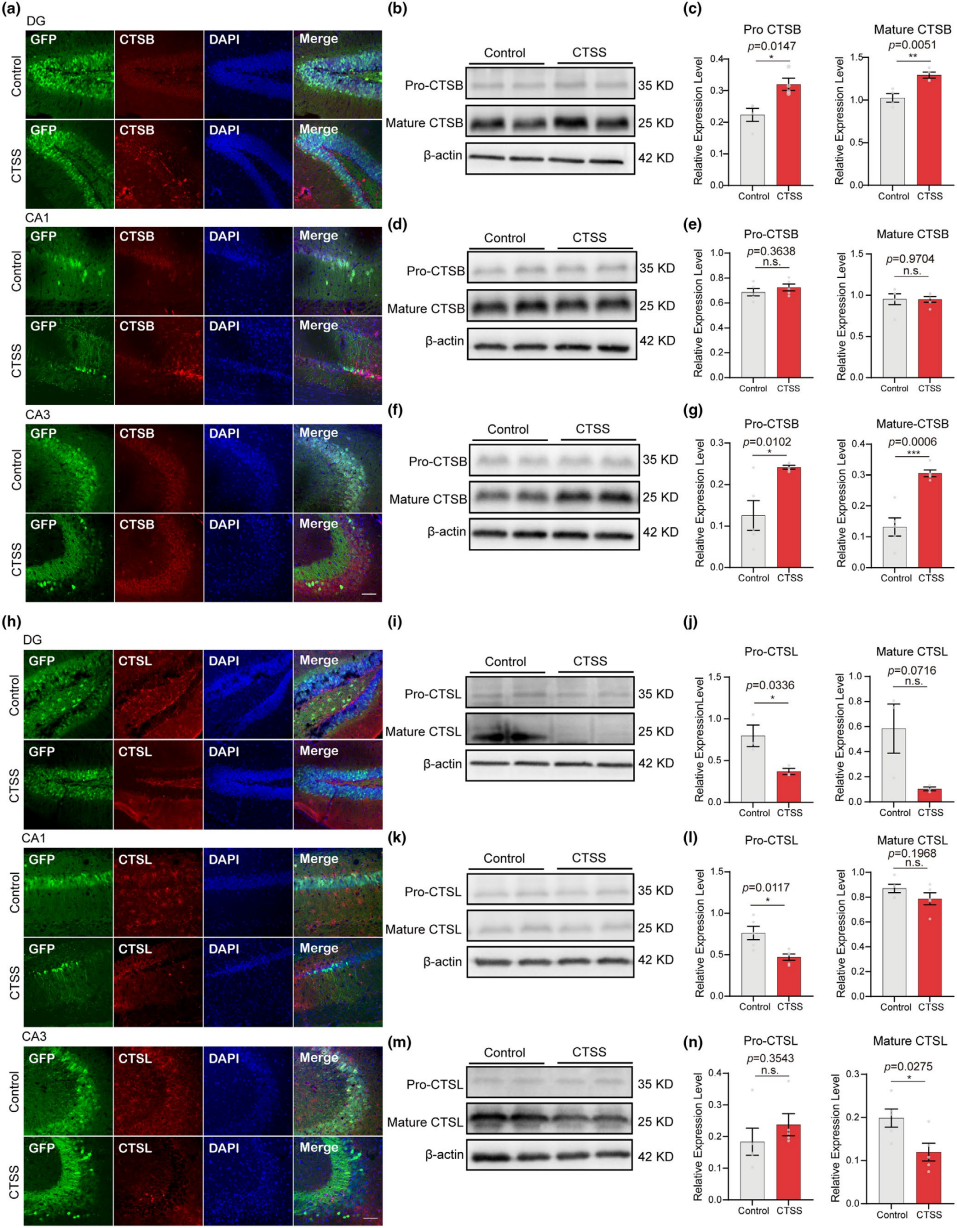
Neuronal CTSS overexpression increased cathepsin B (CTSB) activity, but decreased cathepsin L (CTSL) activity in microglia. (a) Immunostaining of CTSB (red), GFP (green), and DAPI (blue) in the DG, CA1, and CA3 subregions in control and CTSS overexpressed groups. Scale bar: 20 μm. (b) Immunoblot of pro‐CTSB and mature CTSB in the hippocampus of two groups. (c) Immunoblot analysis of the protein levels of pro‐CTSB (left) and mature CTSB (right) in the hippocampus of two groups (Control: *N* = 4, CTSS: *N* = 4). (d) Immunoblot of pro‐CTSB and mature CTSB in HT‐22 cells of two groups. (e) Immunoblot analysis of the protein levels of pro‐CTSB (left) and mature CTSB (right) in HT‐22 cells of two groups (Control: *N* = 5, CTSS: *N* = 5). (f) Immunoblot of pro‐CTSB and mature CTSB in BV2 cells of two groups. (g) Immunoblot analysis of the protein levels of pro‐CTSB (left) and mature CTSB (right) in BV2 cells of two groups (Control: *N* = 5, CTSS: *N* = 5). (h) Immunostaining of CTSL (red), GFP (green), and DAPI (blue) in the DG, CA1, and CA3 subregions in control and CTSS overexpressed groups. Scale bar: 20 μm. (i) Immunoblot of pro‐CTSL and mature CTSL in the hippocampus of two groups. (j) Immunoblot analysis of the protein levels of pro‐CTSL (left) and mature CTSL (right) in the hippocampus of two groups (Control: *N* = 3, CTSS: *N* = 3). (k) Immunoblot of pro‐CTSL and mature CTSL in HT‐22 cells of two groups. (l) Immunoblot analysis of the protein levels of pro‐CTSL (left) and mature CTSL (right) in HT‐22 cells of two groups (Control: *N* = 5, CTSS: N = 5). (m) Immunoblot of pro‐CTSL and mature CTSL in BV2 cells of two groups. (n) Immunoblot analysis of the protein levels of pro‐CTSL (left) and mature CTSL (right) in BV2 cells of two groups (Control: *N* = 5, CTSS: *N* = 5). T‐test was used for (c, e, g, j, l, and n) Data are presented as mean ± SEM. *: *p* < 0.05, **: *p* < 0.01.

CTSS is not the only cysteine cathepsin implicated in inflammation processes and lysosomal membrane permeabilization. CTSB, a lysosomal cysteine protease, undergoes maturation through an autocatalytic deactivation process, and plays a role in the immune system by participating in antigen processing and presentation (Liu et al., 2024). In our study, we initially investigated the impact of neuronal CTSS overexpression on CTSB expression. By immunofluorescence, we observed a significant enhancement in the fluorescence intensity of CTSB within the CA1, CA3, and DG subregions of the hippocampus due to neuronal CTSS overexpression **(**Figure 7a **)**. Studies have shown that CTSB is processed from its proenzyme form to a mature enzyme through a series of proteolytic cleavages facilitated by the acidic environment of lysosomes, which is pivotal for its catalytic activity and physiological roles. We further assessed the protein level of CTSB using immunoblot and discovered an increase in both the precursor and mature forms of CTSB. Since post translational changes in cathepsins may affect the activity and not the protein level (Nagakannan & Eftekharpour, 2017), our result indicated that neuronal CTSS overexpression may elevate CTSB activity to some extent (Figure 7b,c). Subsequently, we applied a coculture system to further verify the cell specificity. The results showed that neuronal CTSS overexpression did not affect the expression levels of precursor and mature CTSB in HT‐22 cells (Figure 7d,e), but significantly increased the expression levels of precursor and mature CTSB in BV2 cells (Figure 7f,g). These findings corroborate the immunofluorescence results, suggesting that neuronal CTSS overexpression increased CTSB protein level specifically in microglia.

Concurrently, we examined the impact of neuronal CTSS overexpression on CTSL expression. CTSL is a lysosomal cysteine protease that matures through a series of proteolytic events, and upregulation of cathepsin L is an important process in the induction of nuclear lamina damage, which is associated with epigenetic modifications in AD pathophysiology (Islam et al., 2022). Unlike the expression pattern observed for CTSB, we found that the fluorescence phenotype of CTSL exhibited a distinct microglial morphology in the control group, whereas neuronal CTSS overexpression significantly reduced the fluorescence intensity of CTSL in the CA1, CA1, and DG subregions of the hippocampus (Figure 7h). Additionally, the expression levels of both the precursor and mature forms of CTSL were decreased, indicating a downregulation of CTSL activity (Figure 7i,j). Utilizing an in vitro coculture system, we detected the expression levels of CTSL in HT‐22 and BV2 cells following neuronal CTSS overexpression. The results indicated that the expression level of precursor CTSL was significantly reduced in HT‐22 cells, while the mature form remained unaffected (Figure 7k,l). In contrast, in BV2 cells, the precursor CTSL level remained constant, yet the mature CTSL was significantly downregulated (Figure 7m,n). These findings are consistent with fluorescence results, suggesting that overexpression of CTSS in neurons significantly reduced CTSL protein levels in microglia.

In summary, our findings demonstrated that neuronal CTSS overexpression increased CTSB protein level, but decreased CTSL protein level in microglia. These observations suggested that CTSS may exert a role in brain aging and AD‐related phenotypes by affecting other cathepsin family members in microglia, including CTSB and CTSL. This insight provides additional mechanism for the role of CTSS, which will be further studied in the future.

## 4 DISCUSSION

Human aging is an incredibly complex process characterized by time‐dependent functional decline, resulting in reduced overall quality of life (Campisi et al., 2019). Notably, the global population over 65 years is growing faster than any other age group. Moreover, with this trend, the United Nations predicts that by 2050, one in six people will be over 65, and the number of people over 80 will triple (Paul & Candelario‐Jalil, 2021). Aging is a predominant risk factor for most chronic diseases in humans (Kennedy et al., 2014) (Hou et al., 2019). Among the different age‐related diseases, neurodegeneration and the associated cognitive decline are particularly relevant owing to their great influence on health and quality of life (Checkoway et al., 2011). However, neither extremely effective nor potent preventive techniques have been developed.

### 4.1 Biomarkers for aging and AD

Studies have defined a series of novel biomarkers of aging with clinical translational value through analyses of multi‐parameter phenomics, single‐cell transcriptomes, proteomes, metabolomes and microbiomes (Aunan et al., 2016). Wang et.al found that plasma sCD22 can be used as one of the biomarkers for evaluating aging and dementia as the sCD22 levels in the plasma of patients with preclinical AD and AD dementia are increased and negatively correlated with cognitive function scores (Bu et al., 2022). c study from Liu et.al reveal for the first time that Endogenous Retrovirus (ERV) reactivation caused by nuclear lamina damage can serve as a biomarker of human frontal aging (Zhang, Li, et al., 2023). Metabolic abnormalities are common in the process of brain aging, and some metabolic biomarkers, such as glucose transporter 1 (GLUT1), some key enzymes involved in glycolysis and oxidative phosphorylation, NAD, and NADH, etc., are believed to be related to brain aging (Mattson & Arumugam, 2018). These biomarkers provide evidence for the early diagnosis of brain aging and aging‐related disease.

**FIGURE 8:**
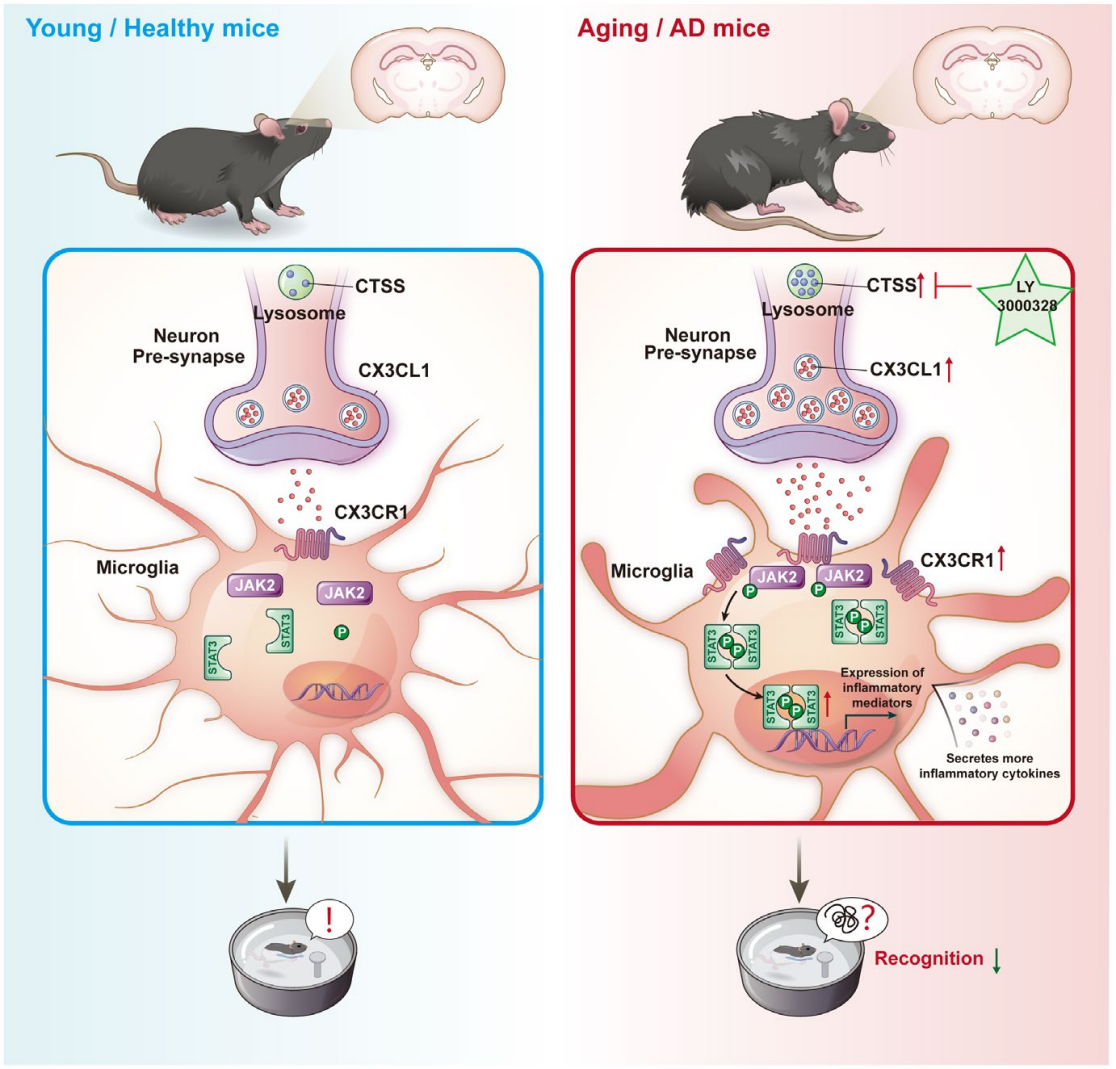
A schematic image shows the role of neuronal CTSS in the process of aging and Alzheimer's disease (AD). Left: In young and healthy mice, neuronal CTSS can induce the secretion of CX3CL1 by neurons and acts on CX3CR1 receptors in microglia, which degrades proteins along the endocytic pathway; Right: In aging and AD model mice, the expression level of neuronal CTSS was significantly elevated in neurons, which increases neuroinflammation and causes cognitive decline via CX3CL1‐CX3CR1 axis and JAK2‐STAT3 pathway. LY 3000328, the selective inhibitor of CTSS, LY3000328, significantly rescues AD‐related pathological features in *APP/PS1* mice.

In our study, we observed a positive correlation between CTSS expression and aging‐related phenotypes, indicating that CTSS could serve as a biomarker for brain aging. After that, we performed a series of behavioral experiments to elucidate the role of neuron CTSS in recognition ability. Our findings revealed that CTSS overexpression in neurons causes spatial learning and memory deficits in young mice. However, CTSS knockdown in neurons rescued the spatial learning and memory deficits in aging mice (Figure 8). Unlike other cysteine cathepsins, CTSS is relatively stable at neutral pH, enabling its action on extracellular substrates beyond its involvement in the endo lysosomal pathway (Wilkinson et al., 2015). Previous studies illustrated that CTSS is preferentially expressed in mononuclear phagocytic cells, like dendritic cells, macrophages, and microglia (Petanceska et al., 1996), (Lin et al., 2021). Moreover, CTSS is vital in adaptive immune responses, contributing to antigen presentation (Yan et al., 2020), (Seo et al., 2016). Our study is the first to establish that CTSS is expressed in neurons and is vital for regulating cognition in mice.

### 4.2 Neuroinflammation in aging and AD

To determine how CTSS affects age‐related phenotypes in mice, we performed RNA‐Seq analysis on the hippocampus of control and CTSS‐overexpressed mice. We found that CTSS overexpression significantly aggravates the brain inflammatory milieu, consistent with its function in the immune response. Studies have shown that aging has been associated with a low‐grade sterile inflammatory status of the immune system, in which pro‐inflammatory cytokines are key players in unhealthy aging (Frungieri et al., 2018). As aging progresses, macrophages and microglia exhibit compromised and prolonged responses to insults, diminished motility, and impaired phagocytosis (Cordano et al., 2022), (Sun et al., 2022). Besides, impaired phagocytosis leads to an augmented accumulation of toxic proteins associated with the progressive pathology of Aβ in AD and α‐synuclein in PD (Wang et al., 2022).

Microglia are the major resident immune cells in the brain, continuously surveying the environment for pathogenic insults, mediating synapse formation and myelogenesis, secreting neurotrophic factors, and phagocytosing debris in the CNS (Prinz et al., 2019). Under physiological conditions, microglia acquire a neural‐specific, relatively inactive phenotype with long cytoplasmic protrusions, stable cell body, and minimal mobility (Borst et al., 2021), (Parajuli & Koizumi, 2023). The maintenance of this inactive state is regulated by intrinsic factors like Runt‐related transcription factor 1 and interferon regulatory factor 8, along with extrinsic factors like triggering receptors also expressed on myeloid cells‐2, chemokine CX3CR1, and CD200R (Kierdorf & Prinz, 2013). Healthy neurons transmit signals to microglia within the normal CNS environment through secreted and membrane‐bound factors, like CX3CL1, neurotransmitters, neurotrophins, and CD22 (Harry, 2013), (Zhao et al., 2021). Upon encountering danger signals, like bacteria or signs of injury, microglia are rapidly activated and migrate toward the injury site. Traditionally, activated microglia can be categorized into two opposing phenotypes: M1 and M2 phenotypes (Ransohoff, 2016). When classically activated, microglia acquire the M1 phenotype, characterized by pro‐inflammatory and pro‐killing functions that serve as the first line of defense. The alternative M2 microglial activation state involves various events, including immunoregulation, inflammation attenuation, and repair and injury resolution facilitation (Oshima et al., 2023). Morphologically, M2 microglia are characterized by enlarged cell bodies. M2 microglial activation produces anti‐inflammatory cytokines, extracellular matrix proteins, glucocorticoids, and other mediators (Dordoe et al., 2023), (Biswas, 2023).

Our results revealed that CTSS overexpression in neurons induced significant activated the microglia with the phenotype switching to M1 and increased release of cytokines, including CD11b, CD86, and CCL5. Subsequently, IF and Immunoblot assays were performed to detect changes in the downstream signaling pathways. We found that neuron CTSS overexpression activated the CX3CL1‐CX3CR1 axis and JAK2‐STAT3 signaling pathway significantly. Given that CX3CL1 is secreted by neurons and acts on CX3CR1 receptors in microglia, our results reveal the role of neuron CTSS in neuron–microglia “crosstalk” for the first time **(**Figure 8 **)**.

### 4.3 Neuronal CTSS exerts differential modulation on CTSB and CTSL

In this study, we have delved into the intricate interplay between neuronal CTSS and its regulatory influence on the enzymatic activities of two other cysteine cathepsins, CTSB and CTSL. Our results elucidate that overexpression of neuronal CTSS is positively correlated with an upregulation of CTSB expression in microglia. This increase in mature CTSB protein level may reflect the elevated CTSB activity, which could be attributed to the facilitation of proteolytic cleavages within the acidic lysosomal environment, and essential for the maturation and catalytic function of CTSB. Conversely, neuronal CTSS overexpression exerts an inhibitory effect on CTSL protein levels. The distinct microglial morphology observed in the control group was notably altered, along with a reduction in the fluorescence intensity of CTSL in the hippocampal subregions. This was corroborated by the decreased expression levels of both precursor and mature forms of CTSL, suggesting a downregulation of its activity (Figure 7).

The contrasting effects of neuronal CTSS overexpression on CTSB and CTSL protein levels in microglia highlight the complex regulatory mechanisms that govern the balance between these two cathepsins. CTSB, known for its role in antigen processing and presentation, may be implicated in the heightened immune response observed in neurodegenerative conditions. In contrast, the downregulation of CTSL, which also plays a critical role in antigen presentation and T‐cell activation, could potentially attenuate immune responses and contribute to the pathophysiology of neuroinflammation (Honey & Rudensky, 2003). Our study, therefore, may provide significant implications for understanding the molecular underpinnings of brain aging and AD. The distinct modulation of these cathepsins could be a key factor influencing the neuroinflammatory milieu and may offer potential therapeutic targets for modulating immune responses in neurodegenerative disorders. Future investigations will delve into the precise molecular mechanisms by which CTSS modulates CTSB and CTSL activities and their collective impact on neuroinflammatory processes.

### 4.4 CTSS serves as a therapeutic target for AD

There is an urgent need to develop effective treatments for AD (Jucker & Walker, 2023), (Veitch et al., 2024). To explore the role of CTSS in the diagnosis and treatment of AD, we administered the CTSS selective inhibitor LY3000328 to the hippocampus of *APP/PS1* mice. Our findings revealed that LY3000328 treatment markedly alleviated the neuroinflammation response, decreased Aβ deposition, and rescued the learning and memory deficits via the CX3CL1‐CX3CR1 axis and JAK2‐STAT3 pathway in *APP/PS1* mice **(**Figure 8 **)**. This result suggested that LY3000328 might be a potential small molecule for treating AD.

### 4.5 Limitations of the study

In our study, we performed the coculture experiments and revealed for the first time that neuronal CTSS participated in neuron–microglia “crosstalk” via CX3CL1‐CX3CR1 axis for the first time. However, the interaction of CTSS and CX3CL1 should be visualized through more precise methods, such as X‐ray or immune‐electron microscopy. In addition, we observed a perfect colocalization of CTSS with Aβ_1‐42_ accumulation, suggesting a regulatory role for CTSS in the transition or clearance of Aβ_1‐42_. Finally, in the last part, we found that neuronal CTSS affect the CTSB and CTSL activity in microglia, which provides another possible mechanism for the role of CTSS. We will focus on these themes in future work.

## 5 CONCLUSIONS

In summary, our study investigated the role of neuron CTSS in regulating learning and memory in aging and AD model mice and the underlying mechanism. Our data reveals a significant increase in the CTSS expression level in aging individuals, which was negatively correlated with the recognition behavior. CTSS overexpression in hippocampus neurons increased neuroinflammation by modulating the CX3CL1‐CX3CR1 axis and JAK2‐STAT3 pathway. Since CX3CL1 is secreted by neurons and acts on the CX3CR1 in microglia, our results unveiled neuron CTSS's role in neuron–microglia “crosstalk” for the first time. Subsequently, we explored the relationship between CTSS and AD. Through retrospective analysis of AlzData databases (http://www.alzdata.org/), we observed elevated expression of CTSS in multiple brain regions of AD patients, including hippocampus. Furthermore, utilizing selective CTSS inhibitor, LY 3000328, has been shown to ameliorate neuroinflammation response, decrease activation of CX3CL1‐CX3CR1 axis and JAK2‐STAT3 pathway, and rescue AD‐related phenotypes in *APP/PS1* transgenic mice. Furthermore, we found that neuronal CTSS overexpression increased the CTSB activity, but decreased the CTSL activity in microglia, which provides another possible mechanism for the role of CTSS. In conclusion, our research has shown that CTSS is expressed in neurons and can be used as a biomarker for aging for the first time. Overall, we discovered that CTSS plays a regulatory role in aging, neuroinflammation, learning, and memory, making it a viable therapeutic target for the diagnosis and treatment of AD.

## AUTHOR CONTRIBUTIONS

P.P.L. designed the experiments. P.P.L., X.H.L., M.J.R., and M.C. performed the behavioral experiments and contributed to the analysis. P.P.L., R.Z.Y., D.D.W., S.A.L., Y.Y., F.X.Y., and Y.H.G. performed viral injection, brain preparation, imaging, and analysis. M.L.L., M.C., and X.T.L. performed the analysis of the RNA‐Seq data. P.P.L., B.H., Y.J.X., J.S.K. and K.D.R. supervised the project. P.P.L. wrote the manuscript. All authors reviewed the manuscript.

## FUNDING INFORMATION

This study was funded by the National Natural Science Foundation of China (grant no. 32000522, 32,000,855, and 82,204,389), the Natural Science Foundation of Henan Province (grant no. 202300410420, and 212,300,410,274), the Joint Construction Project of Henan Province (grant no. 2018020088), the Medical Science and Technology Research Project of Henan Province (grant no. SBGJ202103079), Henan Province Key Research and development and promotion special (Science and technology research) (grant no. 242102311036), and Guangdong Basic and Applied Basic Research Foundation (grant no. 2023B1515230008).

## CONFLICT OF INTEREST STATEMENT

The authors have declared that no conflicts of interest exist.

## Supporting information


Figure S1.
Figure S2.
Figure S3.
Figure S4.
Figure S5.
Figure S6.
Figure S7.
Figure S8.

Table S1.

Table S2.

## Data Availability

The data are provided within the manuscript or supplementary information files. Besides, all original data will be available upon contacting Pei‐Pei Liu (fccliupp@zzu.edu.cn).
