# Supplementary material for: Neuronal cathepsin S increases neuroinflammation and causes cognitive decline via CX3CL1‐CX3CR1 axis and JAK2‐STAT3 pathway in aging and Alzheimer's disease
Source: Aging Cell. 2024 Oct 25;24(2):e14393. doi: 10.1111/acel.14393 (PMC11822647; doi:10.1111/acel.14393)
Supplement: Supplementary file 1 — Figure S1. Figure S2. Figure S3. Figure S4. Figure S5. Figure S6. Figure S7. Figure S8. [file ACEL-24-e14393-s003.docx]

## SUPPLEMENTAL FIGURES


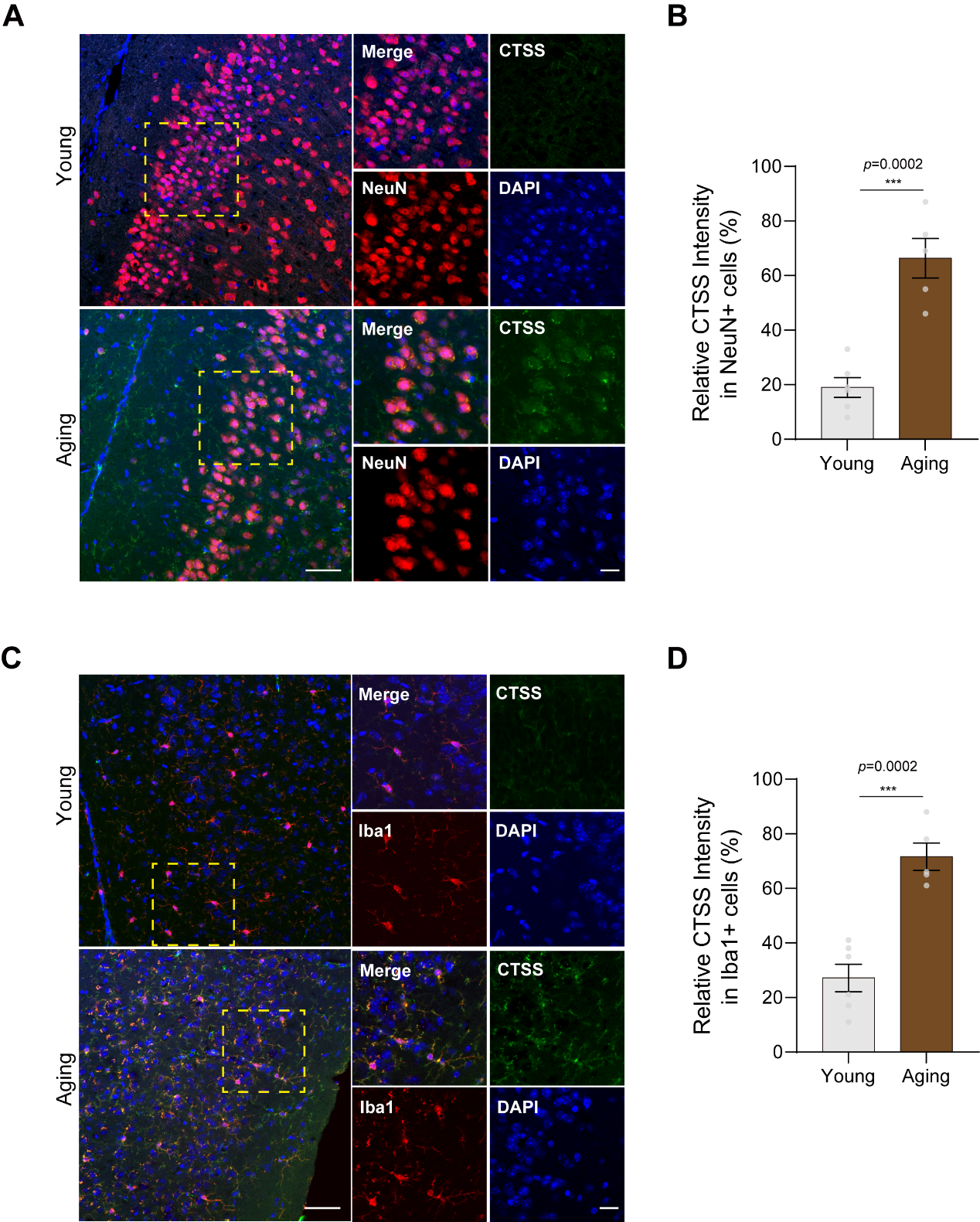


**Supplemental Figure 1. The fluorescence intensity of CTSS both in the neurons and microglia was higher in the aging mice than the young ones in the prefrontal cortex.**

**A.** IF showing that CTSS is colocalized with NeuN in the prefrontal cortex, and the IF intensity of CTSS in NeuN positive cells in aging mice was significantly higher than that in young mice. Scale bar: 20 μm. **B.** Analysis of the relative CTSS intensity in NeuN positive cells in two groups (young: n = 6, aging: n = 5). **C.** IF showing that CTSS is colocalized with Iba1 in the prefrontal cortex, and the IF intensity of CTSS in Iba1 positive cells in aging mice was significantly higher than that in young mice. Scale bar: 20 μm. **D.** Analysis of the relative CTSS intensity in Iba1 positive cells in two groups (young: n = 6, aging: n = 5). A T-test was used for **B** and **D**. Data are presented as mean ± SEM. ****: p < 0.001*.


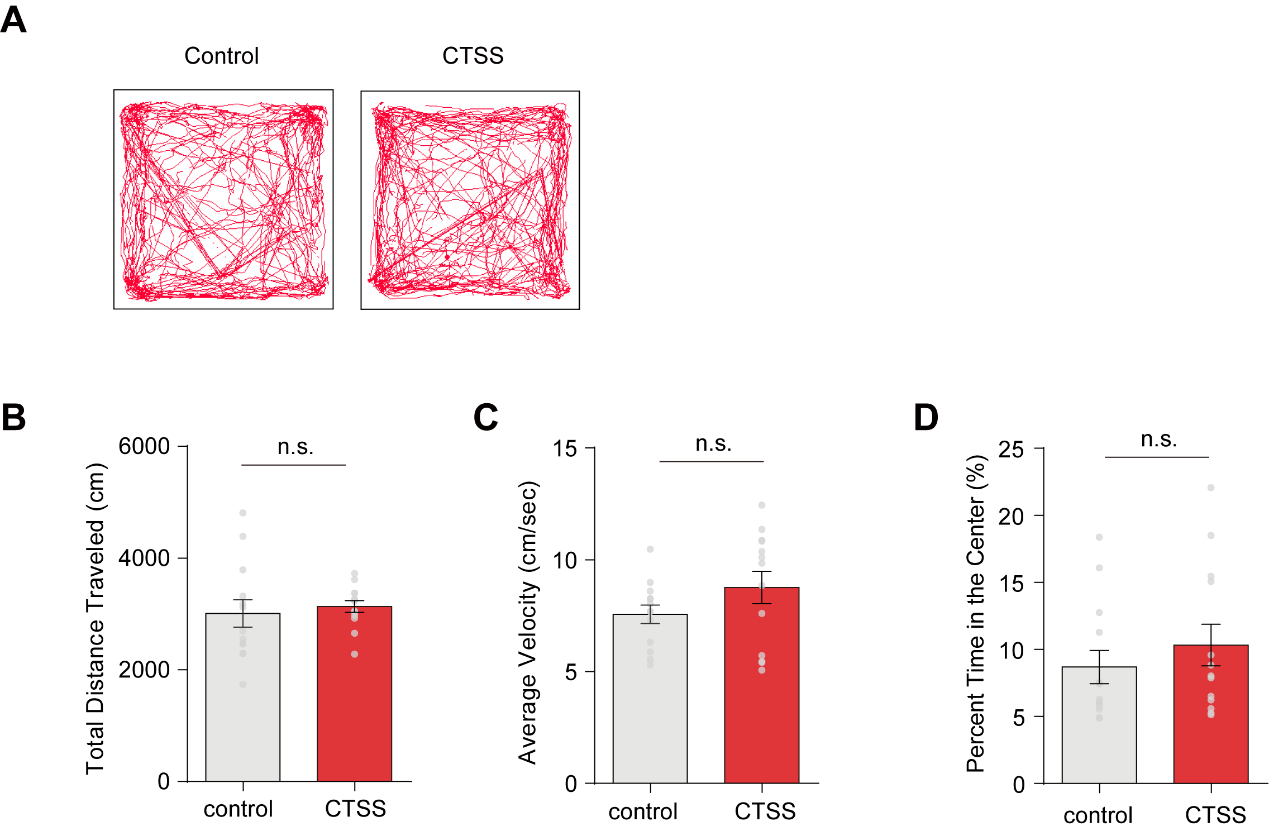


**Supplemental Figure 2. Young mice with CTSS overexpression in hippocampus neurons exhibited normal locomotor activity and anxiety-related behaviors.**

**A.** Trajectory maps of search intensity during open field test. High dwell time is indicated by colors close to red, whereas colors close to blue indicate lower dwell time. **B.** The 10 min total distance traveled is not significantly different between the control and CTSS overexpression groups (n = 13 per group). **C.** The average velocity without resting time is not different between control and CTSS overexpression groups (n = 13 per group). **D.** The percent time in the center is not different between control and CTSS overexpression groups (n = 13 per group). A T-test was used for **B–D**. Data are presented as mean ± SEM.


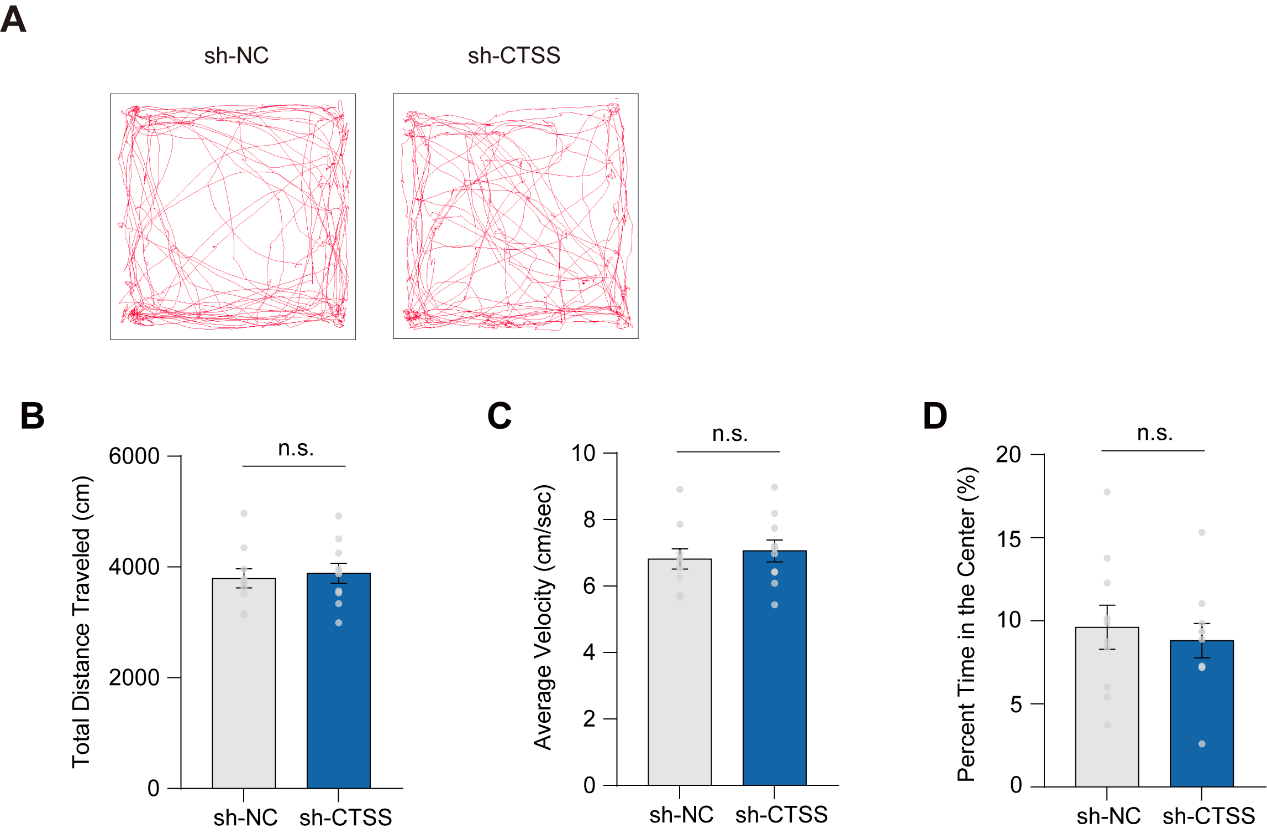


**Supplemental Figure 3. Aging mice with CTSS knockdown in hippocampus neurons exhibited normal locomotor activity and anxiety-related behaviors.**

**A.** Trajectory maps of search intensity during open field test. High dwell time is indicated by colors close to red, whereas colors close to blue indicate lower dwell time. **B.** The 10 min total distance traveled is not significantly different between sh-NC and sh-CTSS groups (n = 10 per group). **C.** The average velocity without resting time is not different between sh-NC and sh-CTSS groups (n = 10 per group). **D.** The percent time in the center is not different between sh-NC and the sh-CTSS groups (n = 10 per group). A T-test was used for **B–D**. Data are presented as mean ± SEM.


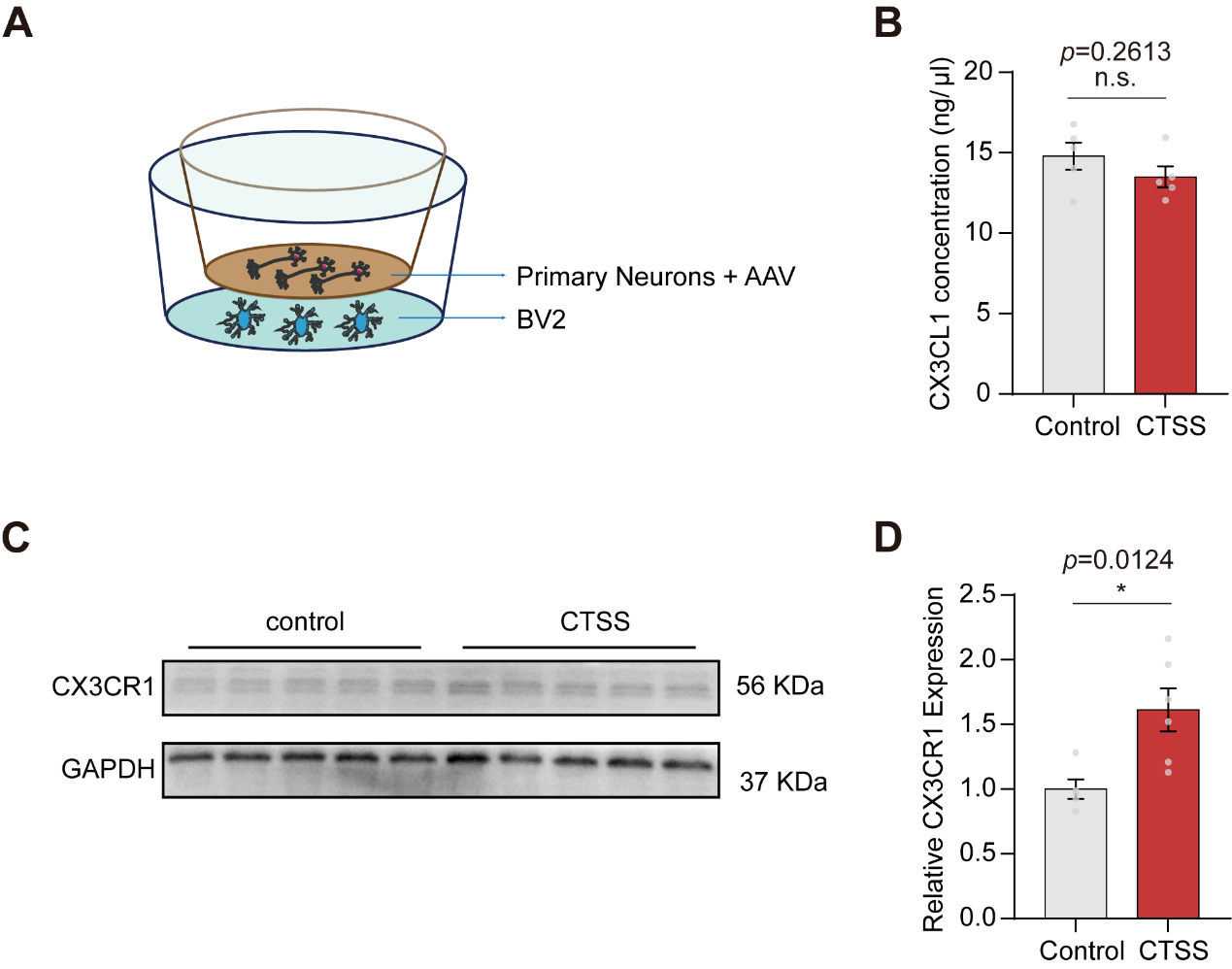


**Supplemental Figure 4. The co-culture assay revealed that overexpression of CTSS in primary neurons did not affect CX3CL1 release, but increased the expression of CX3CR1 in microglia.**

**A.** Illustration of the in vitro co-culture system. The former contains BV2 cells in the lower chamber and primary neurons in the upper chamber. Primary neurons were transfected with either control or CTSS overexpression AAVs for 48h. Co-culture media were collected to measure the concentration of CX3CL1, while BV2 cells were lysed for the detection of CX3CR1 protein level using Immunoblot. **B.** ELISA assay showed that CX3CL1 concentration in the medium of CTSS overexpression group is comparable to that in control group (n = 5 per group). **C.** Immunoblot of CX3CR1 in the two groups. **D.** Immunoblot analysis of the protein levels of CX3CR1 in two groups (control: n = 5, CTSS: n = 6). T-test was used for **B** and **D.** Data are expressed as mean ± SEM. **: p < 0.05*.


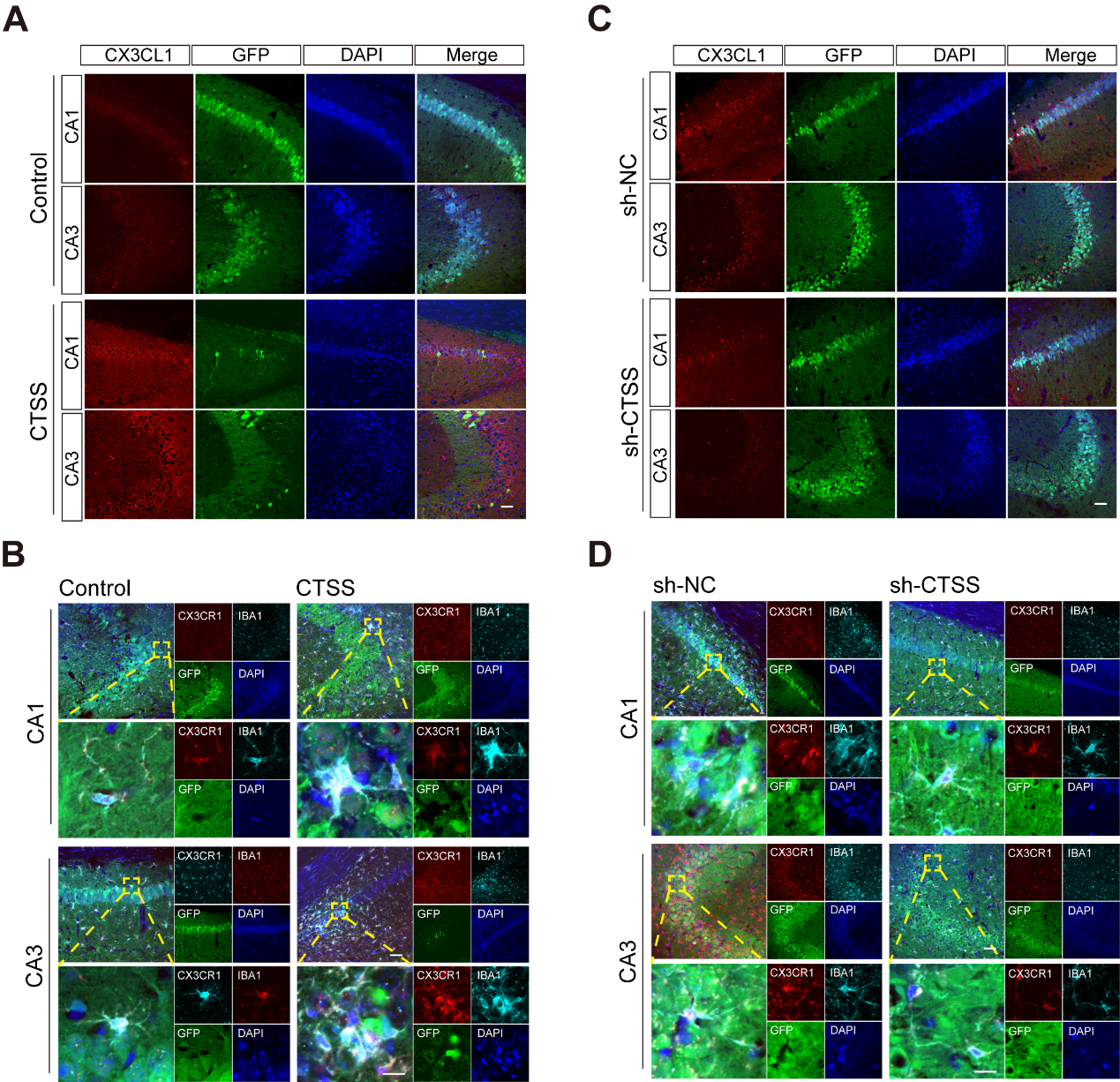


**Supplemental Figure 5. Neuronal CTSS overexpression in young mice elevated the fluorescence intensity of CX3CL1 and CX3CR1, whereas neuronal CTSS knockdown in aging mice decreased the fluorescence intensity of CX3CL1 and CX3CR1.**

**A.** Immunostaining of CX3CL1 (red), GFP (green), and DAPI (blue) in the CA1 and CA3 subregions of hippocampus in control and CTSS overexpressed groups. Scale bar: 20 μm. **B.** Immunostaining of CX3CR1 (red), Iba1 (cyan), GFP (green), and DAPI (blue) in the CA1 and CA3 subregions of hippocampus in control and CTSS overexpressed groups. Scale bar: 20 μm.

**C.** Immunostaining of CX3CL1 (red), GFP (green), and DAPI (blue) in the CA1 and CA3 subregions of hippocampus in NC and CTSS knockdown groups. Scale bar: 20 μm. **D.** Immunostaining of CX3CR1 (red), Iba1 (cyan), GFP (green), and DAPI (blue) in the CA1 and CA3 subregions of hippocampus in NC and CTSS knockdown groups. Scale bar: 20 μm.


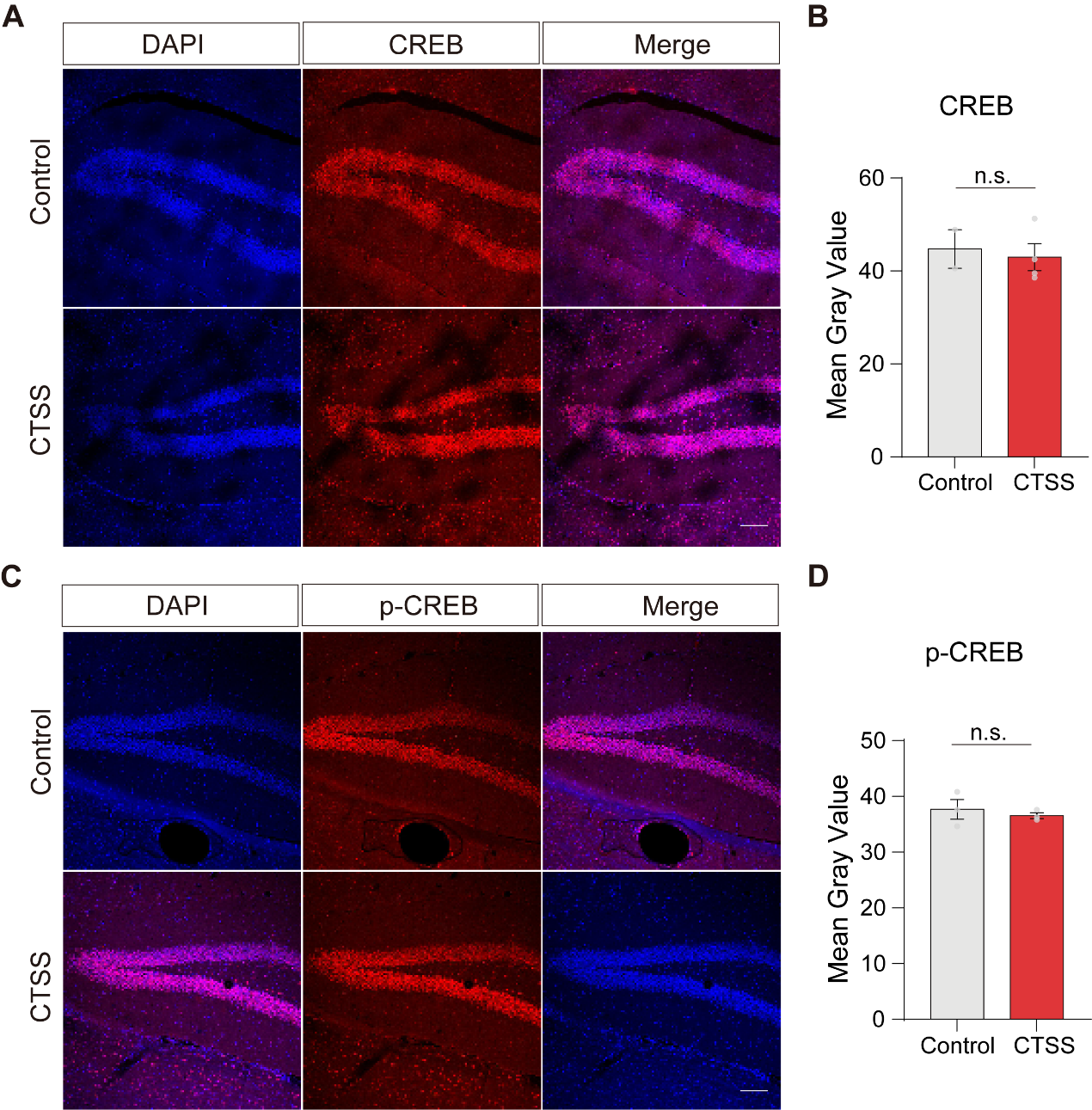


**Supplemental Figure 6. CTSS overexpression in hippocampus neurons of young mice did not affect the expression and phosphorylation levels of CREB.**

**A**. Immunostaining of CREB (red) and DAPI (blue) in the hippocampus of mice in the control and overexpression groups. Scale bar: 50 μm. **B**. Quantification of CREB intensity in the hippocampi of mice in the control and overexpression groups (control: n = 2; CTSS: n = 4). **C**. Immunostaining of p-CREB (red) and DAPI (blue) in the hippocampus of mice in control and overexpression groups. Scale bar: 50 μm. **D**. Quantification of p-CREB intensity in the hippocampus of mice in control and CTSS overexpression groups (n = 3 per group). T-test was used for **B** and **D.** Data are expressed as mean ± SEM.


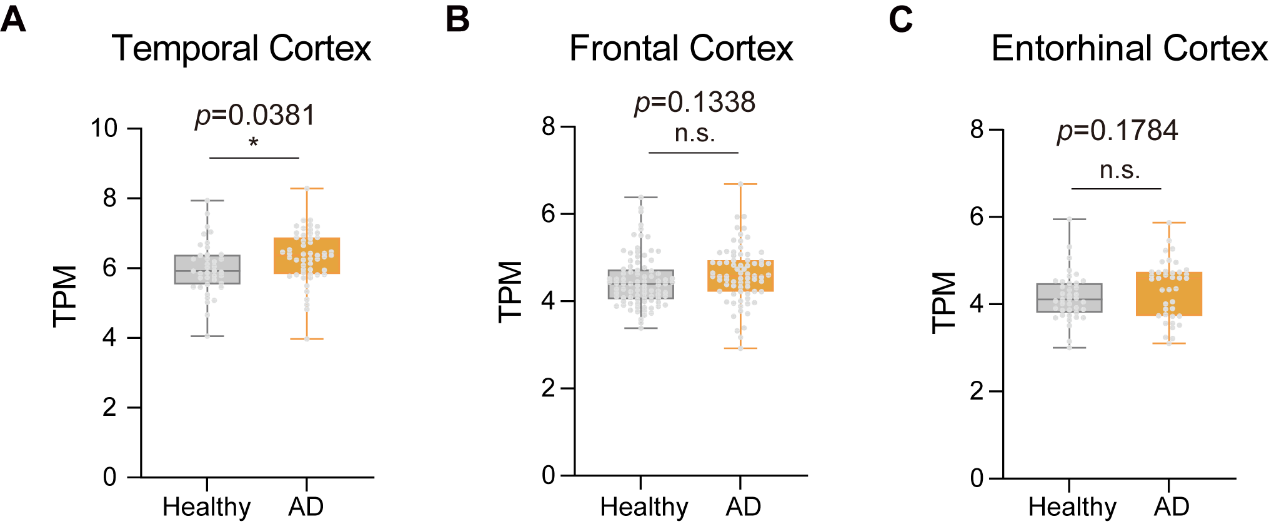


**Supplemental Figure 7. The CTSS Transcripts Per Million (TPM) in the temporal cortex, frontal cortex, and entorhinal cortex of AD patients and their healthy controls.**

**A.** The CTSS TPM in the temporal cortex of AD patients was significantly higher than those in their healthy controls (Healthy: n = 39, AD: n = 52). **B.** The CTSS TPM in the frontal cortex of AD patients was significantly higher than those in their healthy controls (Healthy: n = 88, AD: n = 73). **C.** The CTSS TPM in the entorhinal cortex of AD patients was comparable to those in their healthy controls (Healthy: n = 39, AD: n = 39). T-test was used for **A-C.** Data are expressed as mean ± SEM.


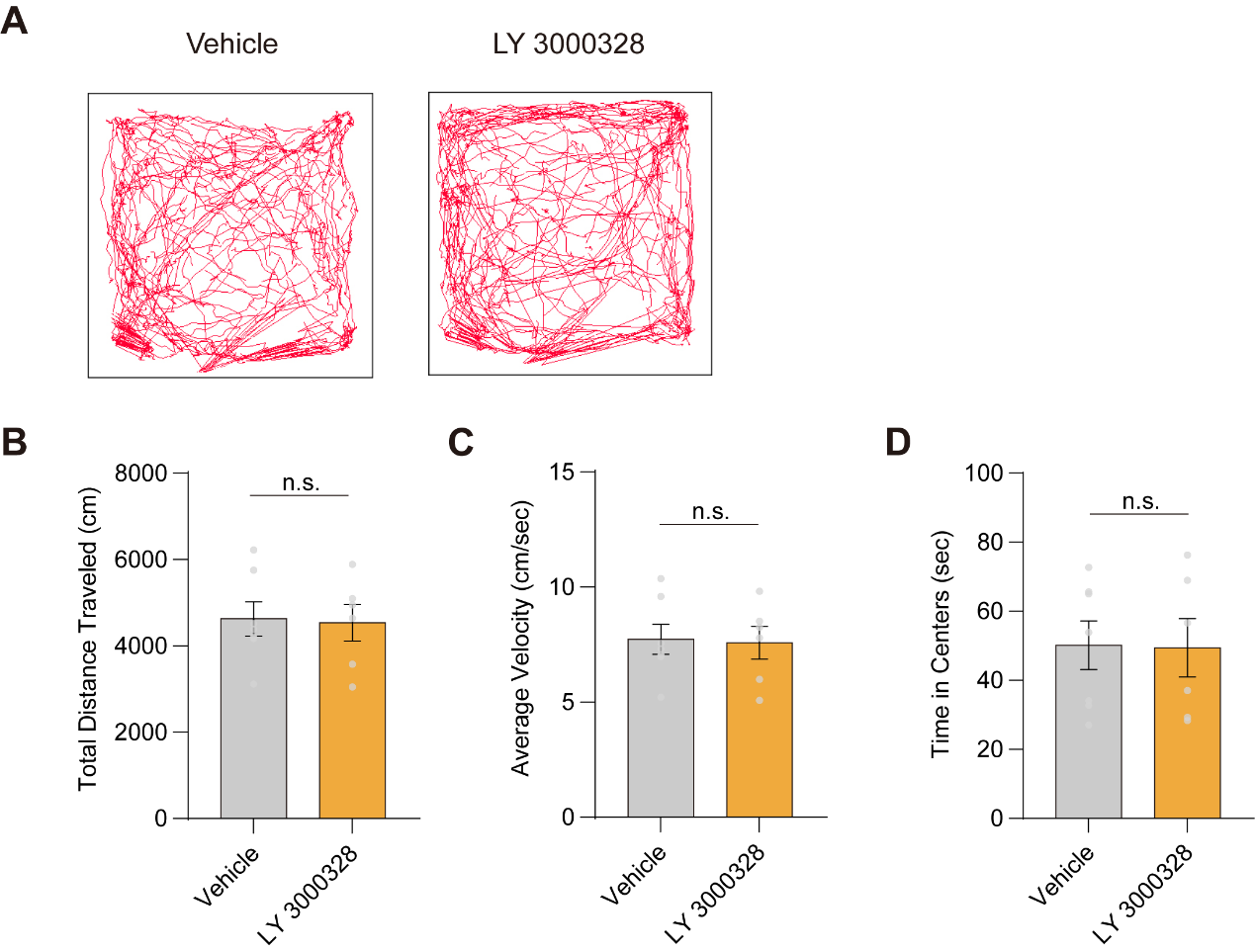


**Supplemental Figure 8. *APP/PS1* transgenic mice in the LY 3000328 group exhibited normal locomotor activity and anxiety-related behaviors compared with the DMSO group.**

**A.** Trajectory maps of search intensity during open field test. High dwell time is indicated by colors close to red, whereas colors close to blue indicate lower dwell time. **B**. The 10 min total distance traveled is not significantly different between the vehicle and LY 3000328 groups (vehicle: n = 7, LY 3000328: n = 6). **C**. The average velocity without resting time is not different between vehicle and LY 3000328 groups (vehicle: n = 7, LY 3000328: n = 6). **D**. The percent time in the center is not different between vehicle and LY 3000328 groups (vehicle: n = 7, LY 3000328: n = 6). T-test was used for **B–D.** Data are presented as mean ± SEM.
